# Discovery of a Novel Tetrapeptide against Influenza A Virus: Rational Design, Synthesis, Bioactivity Evaluation and Computational Studies

**DOI:** 10.3390/ph14100959

**Published:** 2021-09-23

**Authors:** Maria Carmina Scala, Mariangela Agamennone, Agostina Pietrantoni, Veronica Di Sarno, Alessia Bertamino, Fabiana Superti, Pietro Campiglia, Marina Sala

**Affiliations:** 1 Department of Pharmacy, University of Salerno, Via Giovanni Paolo II 132, 84084 Fisciano, Italy; mscala@unisa.it (M.C.S.); vdisarno@unisa.it (V.D.S.); abertamino@unisa.it (A.B.); pcampiglia@unisa.it (P.C.); 2 Department of Pharmacy, University “G. d’Annunzio” of Chieti-Pescara, Via dei Vestini 31, 66100 Chieti, Italy; magamennone@unich.it; 3 National Centre for Innovative Technologies in Public Health, National Institute of Health, Viale Regina Elena 299, 00161 Rome, Italy; agostina.pietrantoni@iss.it (A.P.); fabiana.superti@iss.it (F.S.); 4 Core Facilities, National Institute of Health, Viale Regina Elena 299, 00161 Rome, Italy

**Keywords:** influenza A virus, lactoferrin, tetrapeptides, biophysics, antiviral agents, hemagglutinin

## Abstract

Influenza is a highly contagious, acute respiratory illness, which represents one of the main health issues worldwide. Even though some antivirals are available, the alarming increase in virus strains resistant to them highlights the need to find new drugs. Previously, Superti et al. deeply investigated the mechanism of the anti-influenza virus effect of bovine lactoferrin (bLf) and the role of its tryptic fragments (the N- and C-lobes) in antiviral activity. Recently, through a truncation library, we identified the tetrapeptides, Ac-SKHS-NH_2_ (**1**) and Ac-SLDC-NH_2_ (**2**), derived from bLf C-lobe fragment 418–429, which were able to bind hemagglutinin (HA) and inhibit cell infection in a concentration range of femto- to picomolar. Starting from these results, in this work, we initiated a systematic SAR study on the peptides mentioned above, through an alanine scanning approach. We carried out binding affinity measurements by microscale thermophoresis (MST) and surface plasmon resonance (SPR), as well as hemagglutination inhibition (HI) and virus neutralization (NT) assays on synthesized peptides. Computational studies were performed to identify possible ligand–HA interactions. Results obtained led to the identification of an interesting peptide endowed with broad anti-influenza activity and able to inhibit viral infection to a greater extent of reference peptide.

## 1. Introduction

Influenza is a highly contagious, acute respiratory illness, which represents one of the most important health issues worldwide. There are three types of influenza viruses that infect humans: A, B and C. Influenza A viruses (IAV) also naturally infect a variety of other animal species and are the only influenza viruses known to cause influenza pandemics, which are global epidemics of influenza diseases [1]. The recent outbreak of SARS-CoV-2 dramatically demonstrated the risks of a global viral spread and highlighted the key role of prevention. In this context, influenza A virus is a highly monitored pathogen. It is largely diffused in the avian population, and its spillover to humans could represent a serious threat [2]. Despite its global diffusion, just a few drugs are used by clinics, with vaccination representing the main strategy for preventing infections. Efforts to prevent influenza by vaccination are made difficult by the virus’s ability to rapidly mutate and recombine into antigenically new viral particles, sometimes leading to the emergence of a totally new viral strain. For this reason, at present, the development of antiviral drugs represents a crucial strategy in the control and prevention of seasonal and pandemic influenza infections [3]. Three classes of antiviral drugs have been approved for treatment and prophylaxis of influenza [4]: the adamantane derivatives (amantadine and rimantadine), potent M2 channel blockers [5], neuraminidase inhibitors (NAIs: zanamivir, oseltamivir laninamivir and peramivir) [6,7] and the selective inhibitor of influenza cap-dependent endonuclease (baloxavir marboxil) [8]. However, the capability of viruses to mutate the target proteins represents an obstacle to efficient treatment with these drugs. Based on the above considerations, the need for new compounds against influenza virus able to overcome the disadvantages of the known therapies is evident [9,10].

**Figure 1:**
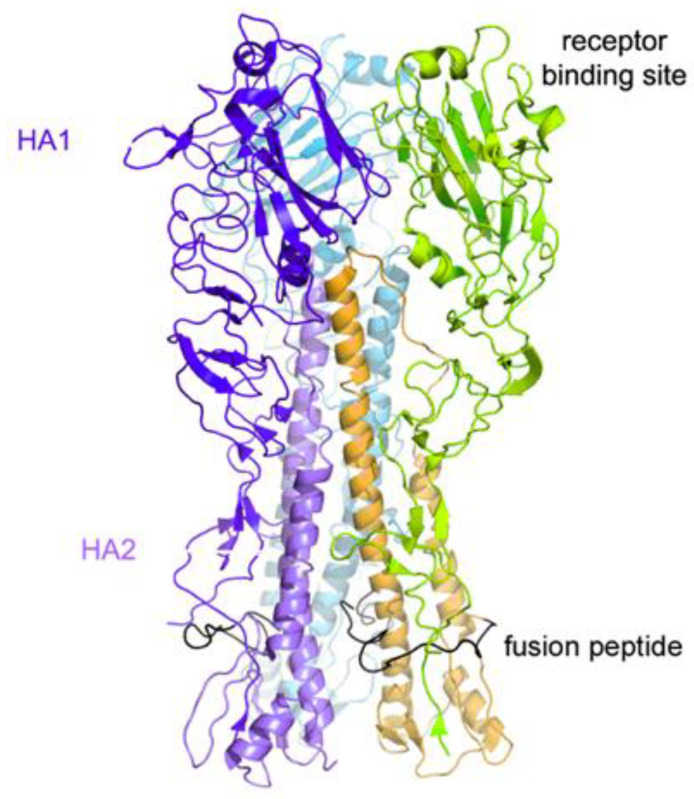
Cartoon representation of the HA trimeric structure.

An attractive antiviral strategy is the blocking of influenza virus entry into the host cell. This process is mediated by viral hemagglutinin. HA is the major surface protein of IAV and is essential to the entry process, thus representing an attractive target for antiviral therapy. It is a large, homotrimeric, mushroom-shaped glycoprotein responsible for initial attachment to the host cell through the receptor-binding site (RBS) and successive viral internalization through membrane fusion promoted by structural rearrangement of the conserved stem region of HA (Figure 1). As a matter of fact, neutralizing compounds targeting HA represents a useful tool in neutralizing viral infection. One notable difficulty in targeting HA is related to its sequence variability among different strains (18 HAs have been identified so far) [11].

Previously, Superti et al. deeply investigated the mechanism of the anti-influenza virus effect of bovine lactoferrin (bLf) and the role of its tryptic fragments (the N- and C-lobes) in antiviral activity [12]. In particular, they evaluated the influence of bLf on hemagglutinin-mediated functions [13].

Recently, through a truncation library, we identified the tetrapeptides Ac-SKHS-NH_2_ (**1**) and Ac-SLDC-NH_2_ (**2**) derived from bLf C-lobe fragment 418–429, which were able to bind HA and inhibit cell infection [14]. By using this information, we further investigated the role of key residues of both peptides in the interaction with HA. This will allow better defining the influence of the chemical modifications introduced in the peptides on their biological properties in terms of affinity and activity. Direct binding assays, bioactivity profile and computational studies led to the identification of a very potent and broad-spectrum tetrapeptide.

## 2. Results and Discussion

### 2.1. Design and Synthesis

The contribution of the various amino acid residues to peptides **1** and **2** activity was further established through L-Ala scanning analysis [15]. This approach allows determining the contribution of side chains of each amino acid residue in the interaction with the target molecule, hemagglutinin. This approach resulted in the generation of a panel of eight peptides (peptides **3**–**10**, Table 1).

Peptides were synthesized according to the solid-phase approach using standard Fmoc methodology in a manual reaction vessel (Material and Methods (Section 3)). The peptides’ purification was achieved using a preparative RP-HPLC C-18 bonded silica column. The purified peptides were 98% pure, as determined by analytical RP-HPLC. The correct molecular weight of the peptides was confirmed by mass spectrometry and amino acid analysis (Table S1, Figures S1–S8, Supplementary Materials).

### 2.2. Direct Binding Assays

**Figure 2:**
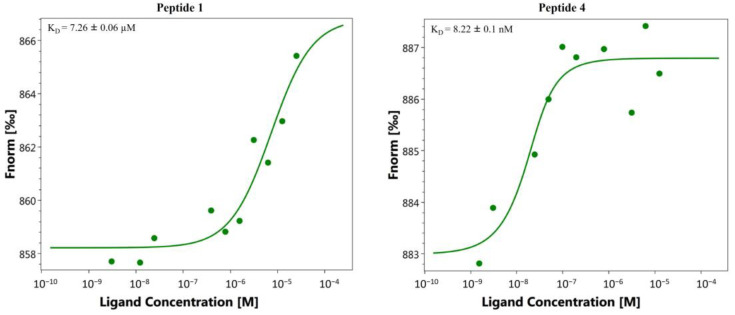
Direct binding measurements of the interaction between HA (H1N1 hemagglutinin, Cat No.: Z03181, GenScript, NE) and peptide **1** and **4**. MST binding affinity assays were performed as described in the Materials and Methods section (Section 3). Representative single dose–response curves of peptides **1** and **4** binding to HA are shown; F_norm_, normalized fluorescence. Experiments were repeated independently three times. Reported K_D_ is the mean ± SD of three independent experiments.

**Figure 3:**
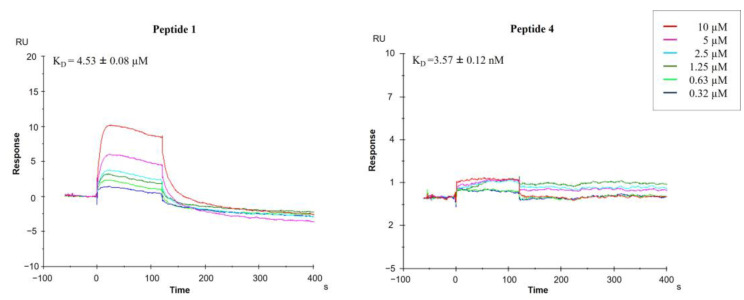
Sensorgrams obtained from the SPR interaction analysis of peptides **1** and **4** binding to immobilized HA (H1N1 hemagglutinin, Cat No: Z03181, GenScript, NE). Each compound was injected at six different concentrations (0.32, 0.63, 1.25, 2.5, 5 and 10 μM). The equilibrium dissociation constants (K_D_) were derived from the ratio between kinetic dissociation (k_off_) and association (k_on_) constants.

There are many biophysical methods available to measure the affinity of ligand–protein interactions. Each technique affords a variety of information on the binding specificity, kinetics thermodynamics and stoichiometry, as well as their advantages and disadvantages, which have been extensively analyzed elsewhere [16]. No single biophysical technique is better than another, as they are based on different principles. For this reason, a more satisfactory approach would be to adopt a strategy that uses one or more orthogonal assays that aim to confirm activity on the target. Based on this consideration, we decided to investigate the direct binding of peptides **1**, **2** and the alanine scanning peptide library to HA protein by two complementary techniques: microscale thermophoresis (Figure 2) and surface plasmon resonance (Figure 3).

#### 2.2.1. Microscale Thermophoresis (MST)

MST is performed using thin capillaries in free solution, which are illuminated with an infrared laser that generates a temperature gradient. The directed movement of molecules is detected by intrinsic fluorescence, or, in most cases, fluorescent labels of one interactant, and quantified. The thermophoretic movement of molecules within the temperature gradient depends on size, charge, hydration shell or conformation that typically changes upon interaction. The thermophoresis signal is plotted against the ligand concentration to obtain a dose–response curve from which the binding affinity can be deduced [17].

MST screening of the 10 compounds was performed as detailed in the Materials and Methods section (Section 3), and results are reported in Table 1. Analysis revealed that all peptides interact with HA with different dissociation constants. In particular, compound **4** binds HA with higher efficiency with respect to **1** (Figure 2), showing an equilibrium dissociation constant (K_D_) value of 8.22 ± 0.1 nM and 7.26 ± 0.06 µM, respectively. MST binding curves of peptides **2**, **3** and **5**–**10** are reported in the Supplementary Materials (Figures S16–S19).

#### 2.2.2. Surface Plasmon Resonance (SPR)

For the SPR study, full-length recombinant HA (His-Tag) was immobilized on a sensor chip up to ~12,000 response units (RU) (Materials and Methods (Section 3)). Compound binding induced a change in the refractive index on the sensor surface [18]. A regeneration step was necessary (glycine, pH 1.5, data not shown). After injection, running buffer was allowed to flow over the surface, and the dissociation of compounds from the surface was observed. In contrast, the control flow cell, where no HA was immobilized, showed no significant signal changes (data not shown). The ability of the tetrapeptides to bind HA was defined by their K_D_ values (Figures S9–S15, Supplementary Materials).

SPR analysis showed the synthesized peptides efficiently interacting with the immobilized protein. Figure 3 presents the sensorgrams of compounds **1** and **4** bound to HA in HBS-P buffer. Interestingly, tetrapeptide **4** binds HA with higher efficiency with respect to **1**, showing a K_D_ value of 3.57 ± 0.12 nM and 4.53 ± 0.08 µM, respectively.

Both the direct binding measurements demonstrated specific binding between peptide **4** and HA. K_D_ of the **4**: HA complex was 8.22 ± 0.10 nM in the MST assay and 3.57 ± 0.12 nM in the SPR assay. This is common as the K_D_ values deeply depend on the analysis method and the applied setup.

### 2.3. Antiviral Activity

#### 2.3.1. Hemagglutination Inhibition Assay (HI)

The ability of peptides to inhibit HA activity was assessed by HI. The inhibitory effect of peptides **1** and **2** is reported as a reference. The IAV strains A/Roma-ISS/02/08 H1N1 oseltamivir-sensitive virus, A/Parma/24/09 H1N1 oseltamivir-resistant virus and A/Parma/05/06 H3N2 were used. As shown in Table 1, Ser1, His3 and Ser4 of peptide **1** are key amino acids for the antiviral activity against some of the IAV strains used in the assay. The substitution of Ser1 with an alanine determines the loss of activity against the influenza A/Parma/24/09 H1N1 virus subtype of the corresponding analog **3** compared to reference peptide **1**. The absence of positive charge by substitution of Lys2 with Ala determines a significant increase in inhibitory potency of the corresponding analog **4** compared to reference peptide **1**. Peptide **5** increases the inhibitor potency of influenza A/H1N1 strains, showing that His3 is important for inhibitory activity of the influenza A/H3N2 virus subtype. In derivative **6**, the substitution of a hydroxyl chain (Ser4) with a more lipophilic residue (Ala) induces a dramatic loss of activity against the two different influenza A/Parma virus subtypes, increasing the antiviral potency of the influenza A/Roma-ISS/2/08 A/H1N1 viral strain. The data also showed that the substitution of each amino acidic residue of peptide **2** with an alanine determines the loss of activity against the influenza A/Roma-ISS/2/08 H1N1 virus subtype, increasing the antiviral potency on the other two different A/Parma virus subtypes.

Therefore, only one peptide, **4**, was able to prevent HA activity of all tested viral strains, in particular, this peptide exerted a strong antiviral action, in the femtomolar range, against two viral strains: A/Roma-ISS/02/08 H1N1 and A/Parma /05/06 H3N2.

#### 2.3.2. Neutralization Assay (NT)

Prompted by previous findings, we assessed the ability of peptide **4** to affect virus replication in the Madin–Darby canine kidney (MDCK) cell line by NT. As shown in Table 2, this peptide was able to prevent infection of all tested viruses in a concentration range from about 0.4 fM to 0.9 pM, with a relevant antiviral activity against the oseltamivir-resistant A/H1N1 strain with an EC_50_ value of about 0.4 fM and a very high selectivity index. Notably, this peptide was more active against all flu strains compared not only to reference peptide **1** but also to all peptides we studied, starting from the bovine lactoferrin (bLf) C-lobe to all derived peptides [12,14].

### 2.4. Computational Studies

To obtain more clues on the tetrapeptide interaction with studied HAs, a structure-based computational analysis was carried out. To be more accurate in the binding prediction, the homology models of our HAs were obtained using the Swiss-Model website (https://swissmodel.expasy.org/, accessed on 29 October 2020) on the basis of their nucleotide sequences kindly provided by Dr. Simona Puzelli (Department of Infectious Diseases, ISS, Rome). The obtained model quality was assessed by the QMean and GMQE algorithms (Table S3, Supplementary Materials).

The identification of ligand-binding regions on the HA surface is not trivial because of the large dimension of this trimeric protein and the great variability between and among viral strains. To locate the putative binding sites of our ligands on the HA surface, we evaluated what was suggested from experimental data: HI accounts for the interference with the sialic acid recognition; therefore, the binding in the RBS was evaluated.

The RBS is responsible for the first binding to the sialic acid of glycoproteins on the host cell surface [19]. It represents a relatively conserved region in the receptor-binding domain (RBD) that, on the contrary, is hypervariable to escape host immunity. For this reason, it can provide a useful tool to fight IAV, also because the displacement of the sialic acid is favored by its low K_D_ in the mM range [20].

A better depiction of the binding site was provided by SiteMap calculation [21,22] that confirmed the hydrophilic character of this site. Different activities between the two A/H1N1 strains can be due to few modified amino acids all around the site: in fact, A/Roma-ISS/02/08 H1N1 has a slightly more flexible loop surrounding the site because of the insertion of an additional Ala125 (alignment of A/Roma and A/Parma H1N1 sequences is reported in the Supplementary Materials (Figure S21).

The docking calculations were carried out setting the SP-peptide docking procedure available in Glide [22,23,24,25] aimed to increase the conformational exploration of ligands during the docking process, retrieving up to 100 docked poses. This procedure is particularly useful to have a picture of possible binding modes of very flexible molecules such as our peptides. Because of the large number of resulting geometries, these were clustered to select the most reliable binding pose for each ligand, taking into account both docking score and number of retrieved similar conformations, so the highest scoring ligand geometry of the most populated cluster is reported in the figures.

**Figure 4:**
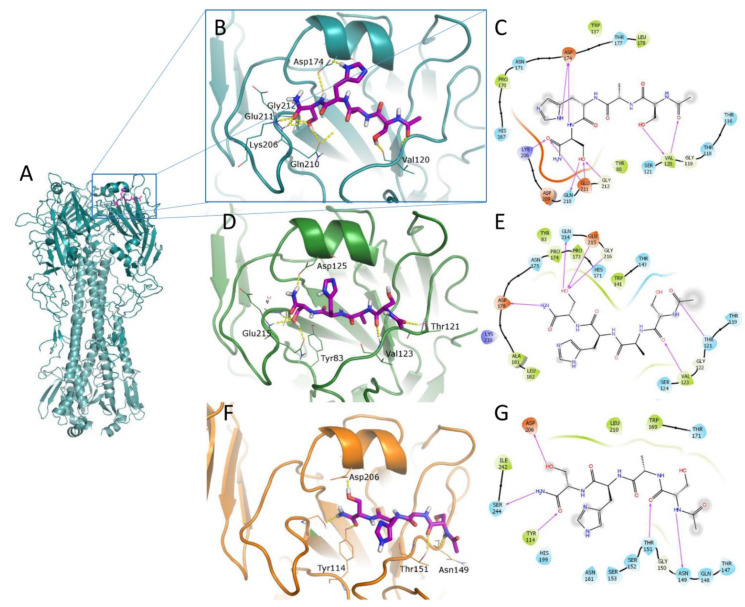
Docked poses of peptide **4** (purple C atoms, represented as sticks) in the RBS of studied HAs represented as cartoons. (**A**) Total view of Parma/H1N1 (deep-cyan HA1 chains, pale cyan HA2 chains); zoomed view of: (**B**) Parma/H1N1 (deep cyan); (**D**) Roma/H1N1 (dark green); (**F**) Parma/H3N2 (orange). Residues involved in H-bond interactions with the ligand are represented as lines; H-bonds are depicted as yellow dashed lines. Corresponding 2D ligand interaction diagrams are reported in panels (**C**,**E**,**G**); in these latter diagrams, residues close to the ligand are colored on the basis of their properties (orange, negatively charged; blue, positively charged; green, hydrophobic; cyan, polar), H-bonds are depicted as magenta arrows, solvent-exposed atoms are surrounded by a gray shadow.

**Figure 5:**
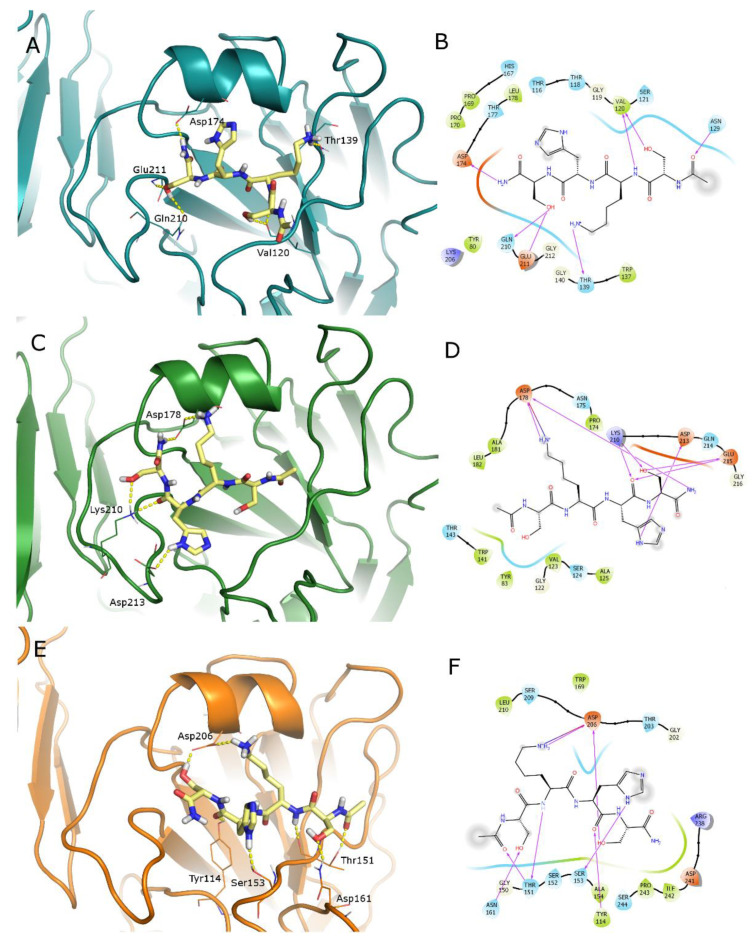
Docked poses of peptide **1** (yellow C atoms, represented as sticks) in the RBS of studied HAs represented as cartoons: (**A**) Parma/H1N1 (deep-cyan); (**C**) Roma/H1N1 (dark green); (**E**) Parma/H3N2 (orange). Residues involved in H-bond interactions with the ligand are represented as lines; H-bonds are depicted as yellow dashed lines. Corresponding 2D ligand interaction diagrams are reported in panels (**B**,**D**,**F**); in the diagrams, residues close to the ligand are colored on the basis of their properties (orange, negatively charged; blue, positively charged; green, hydrophobic; cyan, polar), H-bonds are depicted as magenta arrows, solvent-exposed atoms are surrounded by a gray shadow.

For the sake of simplicity, only the docked poses of the most active compound **4** and reference peptide **1** in the RBS of the three HAs are reported (Figure 4 and Figure 5). A similar binding mode was obtained for the other ligands. Binding geometries of peptide **2** are represented in Figure S22 of the Supplementary Materials as a broad-spectrum representative of the second series of tetrapeptides. The total view of the HA–peptide complex is shown for compound **4** in the Parma/H1N1 (Figure 4A) as a representative. The ligand **4** has a conserved binding geometry in the three studied HAs, spanning the whole site. RBS residues involved in H-bond interactions with our ligands are the same that get into contact with the sialic acid in most cases; in particular, residues corresponding to Asp174, Glu211, Gln210, Tyr80 and Thr118 (A/Parma H1N1 numbering) are responsible for binding with 6′-SLN (PDB ID 3UBN [26]). Most involved ligand residues are the two serine residues forming a network of H-bonds. One of them, in particular, displaces a conserved water molecule with its OH group (Figure S23, Supplementary Materials).

For an explanation of the higher activity observed for peptide **4** compared to its precursor **1**, the docked poses of this latter are reported in Figure 5. The analysis of the retrieved geometries in the RBS does not provide a clear justification: peptide **1** shows a similar occupation of the binding sites with the ligand in an extended conformation and forms a pattern of interactions with the residues engaged with 4. Nevertheless, the long and flexible side chain of Lys residue modifies the binding geometries with respect to peptide **4**.

Some clues can be obtained by evaluating the ligand strain energy, i.e., the energy the ligands spent to reach the binding geometries from its minimum. Data reported in Table 3 suggest that peptide **1** makes a larger effort to reach the docking geometries compared to ligand **4**, probably because of the flexibility of the Lys side chain, which is also reflected in the docking score value. This aspect can contribute to explaining the improved activity of compound **4** with respect to **1**.

## 3. Materials and Methods

### 3.1. Synthesis

Nα-Fmoc-protected amino acids, Rink amide resin, coupling reagents, *N*,*N*-diisopropylethylamine (DIEA), piperidine and trifluoroacetic acid (TFA) were purchased from Iris Biotech (Marktredwitz, Germany). Peptide synthesis solvents, reagents and CH_3_CN for high-performance liquid chromatography (HPLC) were reagent grade, acquired from commercial sources and used without further purification unless otherwise noted.

#### 3.1.1. Peptide Synthesis

The synthesis of tetrapeptides (**1**–**10**) was performed according to the solid-phase approach using standard 9-fluorenylmethoxycarbonyl (Fmoc) methodology [27,28] in a manual reaction vessel on Rink amide resin (0.150 g, 0.7 mmol/g loading) previously Fmoc-deprotected by 25% piperidine solution in DMF (1 × 5 min and 1 × 25 min). Each coupling reaction was accomplished using a 3-fold excess of amino acid with 2-(1H-benzotriazole-1-yl)-1,1,3,3-tetramethyluronium hexafluorophosphate (HBTU) and 1-hydroxybenzotriazole (HOBt) in the presence of DIEA (6 eq.). The peptide resin was washed with dichloromethane (DCM, 3×), *N*,*N*-dimethylformamide (DMF, 3×) and DCM (3×), and the Fmoc deprotection protocol, described above, was repeated after each coupling step. After peptide assembly, the N-terminal Fmoc group was removed, and the peptides were acetylated by adding a solution of Ac_2_O/DCM (1:3) and shaking for 30 min. Finally, peptides were released from the resin using a cleavage mixture containing 90% TFA, 5% triisopropylsilane (TIS) and 5% H_2_O for 2 h. The resin was removed by filtration, and the crude peptide was recovered by precipitation with cold anhydrous ethyl ether to give a white powder and then lyophilized.

#### 3.1.2. Purification and Characterization

All crude peptides were purified by RP-HPLC on a preparative C18-bonded silica column (Phenomenex Kinetex AXIA 100 Å, 100 × 21.2 mm, 5 µm) using a Shimadzu SPD 20 A UV/VIS detector, with detection at 214 and 254 nm. Mobile phase was: (A) H_2_O and (B) ACN, both acidified with 0.1% TFA (*v*/*v*). Injection volume was 5000 µL; flow rate was set to 15 mL/min. The following gradient was employed: 0–18 min, 1–40% B, 18.01–20 min, 40–70% B, 20.01–21 min, 70–90% B, 21.01–23 min, returning to 1% B. Analytical purity and retention time (tr) of each peptide were determined using HPLC conditions in the above solvent system (solvents A and B) programmed at a flow rate of 0.800 mL/min, fitted with a Supelco C-18 column and an Ascentis express peptide C18 column (50 × 3.00 mm, 2.7 µm). LC gradient was the following: 0–7 min, 1–40% B, 7.01–8 min, 40–90% B, 8.01–9 min, returning to 1% B, 9–11 min, isocratic for 2 min. All analogs showed >97% purity when monitored at 220 nm. Homogeneous fractions, as established using analytical HPLC, were pooled and lyophilized.

Ultra-high-resolution mass spectra were obtained by positive ESI infusion on an LTQ Orbitrap XL mass spectrometer (Thermo Scientific, Dreieich, Germany), equipped with Xcalibur software for processing the data acquired. The sample was dissolved in a mixture of water and methanol (50/50) and injected directly into the electrospray source, using a syringe pump, at constant flow (15 µL/min). The temperature of the capillary was set at 220 °C.

### 3.2. Direct Binding Assay

HA was purchased from GenScript, Cat No.: Z03181-100. CM5 sensor chips, HBS-P+ buffer (0.01 M HEPES pH 7.4, 0.15 M NaCl, 0.05% *v*/*v* Surfactant P20), 1-ethyl-3-(3-diaminopropyl) carbodiimide hydrochloride (EDC), N-hydroxysuccinimide (NHS), ethanolamine (H_2_N(CH_2_)_2_OH) and regeneration solution were purchased from Cytiva.

#### 3.2.1. Microscale Thermophoresis (MST)

MST experiments were carried out using the Monolith NT.115pico instrument (NanoTemper Technologies, Munich, Germany). His-HA was labeled using the Nanotemper His-Tag Labeling Kit RED-tris-NTA 2nd Generation as described elsewhere [29]. Briefly, 100 μL of a solution of His-HA protein (80 nM) in double-distilled water was mixed with 100 μL of 40 nM NT647-NHS fluorophore (NanoTemper Technologies, Munich, Germany) in labeling buffer and incubated for 30 min at room temperature. NT647-HA was centrifuged for 10 min at 4 °C at 15,000× *g* to remove protein aggregates. Pretests using standard-treated and premium-coated MST capillaries (NanoTemper Technologies, Munich, Germany) were performed to test the adsorption of labeled HA to capillary walls by analyzing capillary scans recorded by the Monolith NT.115pico prior to MST experiments [30]. The protein did not adsorb to standard-treated capillary walls in assay buffer (PBS). For this reason, the following experiments were performed using a standard-treated capillary. Then, buffer conditions were evaluated to identify the optimal state for MST signal reproducibility and the suppression of unspecific adsorption to capillary walls. Compound stocks (5 mM) in MST buffer were diluted in the assay buffer to reach the highest soluble concentration (50 µM). In MST experiments, 16-fold 1:1 serial dilutions of each compound were mixed with NT647-HA to yield a final reaction volume of 20 µL. After 10 min of incubation at rt, the reaction mixtures were loaded into standard-treated capillaries and subsequently inserted in the chip tray of Monolith NT.115 for thermophoresis analysis and the appraisal of K_D_ values. Signals were recorded at high MST power and 10% LED power. K_D_ values were calculated from compound concentration-dependent changes in normalized fluorescence (F_norm_) of HA after 21s of thermophoresis. Each compound was tested in triplicate and data analyzed using MO Affinity Analysis software (NanoTemper Technologies, Munich, Germany). Confidence values (±) were indicated next to the K_D_ value for each tested compound.

#### 3.2.2. Surface Plasmon Resonance (SPR)

SPR binding studies were performed at 25 °C using Biacore T200 (Cytiva, Uppsala, SwedenHis-HA were stably captured at the surface of the CM5 sensor chip by means of an antihistidine antibody (His Capture Kit, Cytiva) that had been covalently bound to the surface as recommended by the manufacturer. In particular, the antihistidine antibody provided in the His Capture Kit was diluted to 50 µg/mL in the immobilization buffer included in the kit and covalently coupled to Sensor Chip CM5 by standard amine coupling to a level of approximately 12,000 RU. Then, His-HA was injected (21,5 μg·mL^−1^ 10 mm acetate, pH 4.59) over the antihistidine antibody surface for 1 min. No protein was injected over the reference surface. The dissociation was monitored by injecting running buffer for 600 s. Surface regeneration was performed by injecting glycine buffer (10 mM, pH 1.5, 1 min).

HBS-P+ buffer was used as running buffer. After the immobilization of HA, HBS-P+ buffer was injected over the chip at a flow rate of 5 μL/min overnight. A solution of peptide in HBS-P+ buffer at various concentrations (from 0.32 to 10 µM) was injected at 25 °C with a flow rate of 30 μL/min for 120 s (association phase), and then the buffer alone was injected for 600 s (dissociation phase).

The equilibrium dissociation constants (K_D_) and kinetic dissociation (kd) and association (ka) constants were calculated from the sensorgrams by global fitting of a 1:1 binding model using BIAevaluation software (v3.1) provided with the Biacore T200 instrument (Cytiva).

### 3.3. Biological Assay

#### 3.3.1. Cells and Viral Strains

Madin–Darby canine kidney (MDCK, ATCC, CRL-2936) cells were grown at 37 °C in minimal essential medium (MEM, Invitrogen, Paisley, UK) containing 1.2 g/L NaHCO_3_ and supplemented with 10% inactivated fetal calf serum (FCS, Invitrogen, Paisley, UK), 2 mM glutamine, nonessential amino acids, penicillin (100 IU/mL) and streptomycin (100 μg/mL). The following IAV strains were used: A/RomaISS/02/08 H1N1 (Brisbane-like) oseltamivir-sensitive virus, A/Parma/24/09 H1N1 (Brisbane-like) oseltamivir-resistant virus and A/Parma/05/06 H3N2 (Wisconsin-like) virus. Viruses were propagated in MDCK cells in serum-free MEM supplemented with 4% bovine serum albumin (BSA fraction V, Gibco; Paisley, UK), 1 µg/µL N-tosyl-L-phenylalanine chloromethyl ketone-treated trypsin (Sigma Chemical Co.; St. Louis, MO, USA). When an extensive cytopathic effect (c.p.e.) was observed, infected cultures were frozen and thawed three times, centrifuged (3000 rpm, 10 min), and supernatants were stored at −80 °C. Titers of virus stocks were determined by hemagglutinin titration and/or plaque assay according to the standard procedures [31,32].

#### 3.3.2. Cytotoxicity Assay

This procedure was performed as reported elsewhere [33]. Briefly, two-fold serial dilutions of each peptide in culture medium were incubated at 37 °C with confluent MDCK cells grown in 96-well tissue culture microplates (Nalge Nunc Europe Ltd., Neerijse, Belgium). After 24 h, the following parameters were evaluated: cell morphology, which was examined by light microscopy; cell viability, which was determined by neutral red staining as already described by us [34]; and cell proliferation, which was evaluated quantitatively by microscopic counts after dispersion into individual cells with trypsin. Peptide dilutions that did not affect any of these parameters were considered noncytotoxic concentrations and utilized for antiviral assays.

#### 3.3.3. Hemagglutination Inhibition Assay (HI)

Viruses in phosphate-buffered saline (PBS, pH 7.4) were incubated for 1 h at 4 °C with serial dilutions of peptides in PBS. An equal volume of 0.5% turkey erythrocytes was then added and allowed to agglutinate. Titers were expressed as the reciprocal of the peptide dilutions giving 50% hemagglutination of erythrocytes by four virus-agglutinating units.

#### 3.3.4. Neutralization Assay (NT)

NT was carried out by incubating serial two-fold peptide dilutions, starting from 12.5 µM, in culture medium with equal volumes of viral suspension containing 10^6^ plaque-forming units (p.f.u.) for 1 h at 4 °C. In negative controls, culture medium was used instead of peptides in the same volume. MDCK cells, grown in 96-well tissue culture microplates (Nalge Nunc Europe Ltd., Neerijse, Belgium), were infected with 100 μL/well (10 p.f.u./cell; in quadruplicate) of the virus–peptide mixtures. After adsorption, cells were rinsed thoroughly and incubated at 37 °C for 24 h. The viral c.p.e. was measured by neutral red staining as reported elsewhere by our laboratory [33].

### 3.4. Computational Studies

#### 3.4.1. Homology Modeling

Homology models for the three viral strains were generated using the Swiss Model webserver (https://swissmodel.expasy.org/, accessed on 29 October 2020) [35]. The FASTA nucleotide sequence of the three HAs (A/Parma H1N1, A/Roma H1N1, A/Parma H3N2) was used to search for the best-matching proteins. To obtain the trimeric form, the quaternary structure annotation was introduced in the FASTA sequence. Suitable templates were aligned using the Basic Local Alignment Search Tool (BLAST) [36] and Hidden Markov model-based lightning-fast iterative sequence search (HHblits) [37]. The highest-ranking template was selected to build the protein models using ProMod3 [38]. The quality of obtained models was assessed using Qualitative Model Energy ANalysis (QMEAN) [39] and Global Model Quality Estimate (GMQE). The Ramachandran plot for each obtained structure was generated to determine the stereochemical and conformational quality (Figure S20, Supplementary Materials). The validity of the obtained quaternary structure was provided by the Quaternary Structure Quality Estimate (QSQE) score [40]. Identified templates and related score values for the three models are reported in the Supplementary Materials (Tables S2 and S3).

#### 3.4.2. Protein Preparation

The three proteins were aligned to each other in Maestro using the Protein Structure Alignment tool that performs the process of aligning the protein sequences.

Superimposed homology models were submitted to the Protein Preparation routine in Maestro in order to optimize the obtained structures. In particular, the H-bond optimizer and the restrained minimization were carried out to ameliorate the H-bond network.

#### 3.4.3. Binding Site Identification and Analysis

To locate the putative binding site on the receptor-binding domain of the studied HAs, the homology models were aligned to the available X-ray complex of pandemic HA bound to 6′-SLN (PDB ID: 3UBN, res 2.51 Å [26]).

Each putative site was explored by performing a SiteMap [21,22] calculation using the following settings: evaluate a single binding site using the aligned X-ray ligand as a reference. The fine grid and more restrictive definition of hydrophobicity were applied. The site was cropped at 6 Å from the last site point.

#### 3.4.4. Receptor Grid Generation

For all sites mentioned in the previous paragraph, the receptor grid was generated using the Receptor Grid Generation routine available in Glide [22,23,24,25]. The grid box was enlarged to dock ligands ≤20 Å, and the inner box size was set to: *x* = 10, *y* = 15, *z* = 10. All grids were generated with settings suitable to peptide docking.

All sites in the RBD were located exploiting the SiteMap calculated site points The following rotatable groups were set: Thr147, Thr151, Ser152, Ser153, Thr171, Thr203, Ser209 for Parma/H3N2; Tyr80, Ser122, Thr177, Tyr179 for A/Parma H1N1 and Tyr83, Thr121, Se133, Thr143, Tyr183 for Roma/H1N1.

#### 3.4.5. Ligand Preparation

The structures of the studied peptides **1**–**10** were built in Maestro using the Build tool. Obtained structures were submitted to Ligprep to generate possible tautomers and protomers at physiological pH (7.0 ± 0.4). Resulting structures were minimized to a derivative convergence of 0.001 kJ/mol^−1^ using the PRCG minimization algorithm, the OPLS3e force field and the generalized Born/surface area (GB/SA) water solvation model implemented in MacroModel [22].

#### 3.4.6. Docking Calculations

Minimized ligand structures were docked in all the previously identified sites on the HA RBD using Glide and the SP-peptide docking protocol that allows increasing the conformational exploration of ligands, affording a final set of 100 poses. Obtained poses for each ligand were clustered using the Clustering of Conformers tool available in Maestro. The Average clustering algorithm was used on the basis of the RMSD calculated between heavy atom pairs. The Kelley penalty was calculated, and the optimal clustering level was set consequently. Most populated clusters were evaluated along with the docking score. To calculate the docked poses strain energy, the corresponding tool available in Maestro was used that performed a constrained and full minimization of the docked poses of ligands **1** and **4** in the three studied HAs. The default parameters were applied in the calculation.

## 4. Conclusions

In this paper, we performed a systematic SAR study through the development of ala-scan peptides starting from the most promising tetrapeptides identified previously [14]. This study allowed us to assess the importance of the side chain on peptide bioactivity.

Direct binding assays were carried out exploiting two biophysical methods (MST and SPR), while the evaluation of antiviral activity was assessed through both HI and NT studies. Structure-based computational studies allowed us to envision the putative interactions of this ligand with HA, highlighting the role of serine residues in receptor binding. All applied methods agreed upon the identification of a novel potent tetrapeptide, Ac-SAHS-NH_2_, able to bind hemagglutinin with high affinity and inhibit influenza virus hemagglutination and cell infection at femtomolar concentration. This small sequence, with high and broad-spectrum activity, can represent a valuable starting point for the design of small molecules. The work carried out opens the way to new perspectives for the development of new anti-influenza drugs, especially in a context in which the emergence of new and drug-resistant viruses highlights the need for new antiviral approaches and strategies.

## Figures and Tables

**Table 1:** Structure characterization, affinity and activity of peptides **1**–**10**. The HI activity of peptides **1** and **2** is reported as a reference. ^a^ Scala et al. (2017) [14]. All peptides are amidated and acetylated. ^a^ The HI titer, determined by the hemagglutination inhibition assay, corresponds to the concentration of the peptide that produces 50% inhibition of virus-induced hemagglutination, i.e., IC50. ^b^ N.D.: not detectable at the maximum tested concentration (12.5 × 10^3^ nM).

Pep.	Seq.	MST K_D_ (μM)	SPR K_D_ (μM)	HI Titer ^a^ (nM)		
				A/Roma-ISS/02/08 H1N1	A/Parma/24/09 H1N1	A/Parma/05/06 H3N2
**1** ^a^	SKHS	7.26 ± 0.06	4.53 ± 0.08	0.1	1.5	12
**3**	AKHS	3.12 ± 0.11	2.7 ± 0.04	0.6	N.D. ^b^	12
**4**	SAHS	0.0082 ± 0.0001	0.0035 ± 0.00012	1.8 × 10^−6^	0.5	2.4 × 10^−6^
**5**	SKAS	7.01 ± 0.09	1.03 ± 0.01	1.8 × 10^−6^	2.9 × 10^−3^	N.D. ^b^
**6**	SKHA	11.4 ± 0.17	6.75 ± 0.81	5 × 10^−7^	N.D. ^b^	N.D. ^b^
**2** ^a^	SLDC	10.4 ± 0.23	7.12 ± 0.26	1.4 × 10^−6^	6	1.5
**7**	ALDC	21.2 ± 0.41	0.0277 ± 0.0017	N.D. ^b^	9 × 10^−7^	3.6 × 10^−7^
**8**	SADC	6.38 ± 0.21	2.19 ± 0.51	N.D. ^b^	2.2 × 10^−6^	5 × 10^−7^
**9**	SLAC	0.0058 ± 0.0003	2.57 ± 0.34	N.D. ^b^	5 × 10^−7^	9 × 10^−7^
**10**	SLDA	2.69 ± 0.09	0.343 ± 0.019	N.D. ^b^	6.1	2.1 × 10^−3^

**Table 2:** In vitro antiviral activity of peptides **1** and **4** against influenza virus infection. ^a^ EC_50_: the reciprocal substance dilution at which 50% of cells were protected from the virus-induced killing; ^SI: the ratio between CC_50_ (the reciprocal substance dilution at which 50% of cells were protected from substance toxicity, corresponding to a concentration >25 μM) and EC_50_; The mean values of 3 independent experiments with standard errors are shown. ^b^ Scala et al. (2017) [14].

Pep.	Seq.	A/Roma-ISS/02/08 H1N1		A/Parma/24/09 H1N1		A/Parma/05/06 H3N2	
		EC_50_^a^ (μM)	SI	EC_50_^a^ (μM)	SI	EC_50_^a^ (μM)	SI
**1** ^b^	SKHS	3 ± 0.61 × 10^−6^	>8.33 × 10^6^	4.8 ± 0.12 × 10^−8^	>5.2 × 10^8^	5 ± 0.02 × 10^−6^	>5 × 10^6^
**2** ^b^	SLDC	5 ± 0.01 × 10^−7^	>5 × 10^7^	4.6 ± 0.05 × 10^−6^	>5.4 × 10^6^	4.3 ± 0.03 × 10^−6^	>5.8 × 10^7^
**4**	SAHS	5.77 ± 0.01 × 10^−7^	>4.33 × 10^7^	4.3 ± 0.3 × 10^−10^	>5.81 × 10^10^	9.36 ± 0.1 × 10^−7^	>2.67 × 10^7^

**Table 3:** Calculated strain energy and docking score of docked poses of peptides **1** and **4** in the studied HAs. These values are obtained running both a constrained and an unconstrained minimization with MacroModel. The energy difference is used to determine the strain energy.

HA Subtype	Pep.	Strain Energy	Docking Score
Roma/H1N1	**1**	6.682	−5.174
	**4**	2.525	−7.554
Parma/H1N1	**1**	9.014	−5.266
	**4**	4.050	−6.259
Parma/H3N2	**1**	6.958	−5.230
	**4**	2.081	−6.137

## Data Availability

Data are within the article and Supplementary Materials.

## Supplementary Materials

The following are available online at https://www.mdpi.com/article/10.3390/ph14100959/s1, Table S1. Analytical data of peptides **1**–**10**; Figures S1–S8. HRMS spectra and HPLC chromatograms of peptide **3**–**10**; Figures S9–S15. Sensor grams of peptides **3**, **5**–**10**; Figures S16–S19. MST binding curves of peptides **2**, **3**, **5**–**10**; Table S2. Swiss-Model data of homology models; Table S3. Score values obtained for the best homology models; Figure S20. Ramachandran Plots of HA homology models; Figure S21. Alignment of HA sequences of A/Parma and A/Roma H1N1 strains; Figure S22. Docked poses of peptide 2; Figure S23. Superposition of the complex of HA: sialic acid with docked pose of SAHS.
