# Supplementary material for: Discovery of a Novel Tetrapeptide against Influenza A Virus: Rational Design, Synthesis, Bioactivity Evaluation and Computational Studies
Source: Pharmaceuticals (Basel). 2021 Sep 23;14(10):959. doi: 10.3390/ph14100959 (PMC8537277; doi:10.3390/ph14100959)
Supplement: Supplementary file 1 [file pharmaceuticals-14-00959-s001.zip › pharmaceuticals-1341957-supplementary.pdf]

SUPPLEMENTARY MATERIALS

Discovery of a novel tetrapeptide against Influenza A virus: rational design, synthesis, bioactivity evaluation and computational studies.

Maria Carmina Scala[a, §], Mariangela Agamennone[b, §], Agostina Pietrantoni,[c,d] Veronica Di Sarno,[a] Alessia Bertamino,[a] Fabiana Superti[c], Pietro Campiglia[a] and Marina Sala[a,]*

[a]Department of Pharmacy, University of Salerno, Via Giovanni Paolo II 132, 84084, Fisciano (SA), Italy

[b]Department of Pharmacy, University "G. d'Annunzio" of Chieti-Pescara, Via dei Vestini 31, 66100, Chieti, Italy

[c]National Centre for Innovative Technologies in Public Health, National Institute of Health, Viale Regina Elena 299, 00161, Rome, Italy

[d]Core Facilities, National Institute of Health, Viale Regina Elena 299, 00161, Rome, Italy

* Corresponding author: msala@unisa.it; Tel.: 089968148

[§] These authors contributed equally to this work

Table of contents:

Supplementary schemes and figures:

**Table S1:**	Analytical data of peptides **1-10**	S2
**Figure S1-S8:**	HRMS spectra and HPLC chromatograms of peptide **3-10**	S2
**Figure S9-S15:**	Sensorgrams of peptides **3, 5-10**	S10
**Figure S16-S19:**	MST binding curves of peptides **2, 3, 5-10**	S13
**Table S2:**	Swiss-Model data of homology models	S14
**Table S3:**	Score values obtained for the best homology models	S15
**Figure S20:**	Ramachandran Plots of HA homology models	S15
**Figure S21:**	Alignment of HA sequences of A/Parma and A/Roma H1N1 strains	S16
**Figure S22:**	Docked poses of peptide **2**...	S17
**Figure S23:**	Superposition of the complex of HA:sialic acid with docked pose of SAHS ...	S18

Table S1. Analytical data of peptides **3-10**.

**Pep.**	**Sequence**	**HPLC k'[a]**	**HRMS**
**1[a]**	SKHS	4.68	499.26235
**3**	AKHS	1.10	483.2659
**4**	SAHS	0.54	443.1667
**5**	SKAS	1.85	434.2055
**6**	SKHA	4.37	483.2654
**2[a]**	SLDC	3.93	477.18970
**7**	ALDC	5.67	462.2003
**8**	SADC	3.50	436.1484
**9**	SLAC	5.16	434.2050
**10**	SLDA	5.93	446.2235

[a] k'=[(peptide retention time-solvent retention time)/solvent retention time].

Supplementary figures of Mass spectrometry and HPLC of peptides used in the study

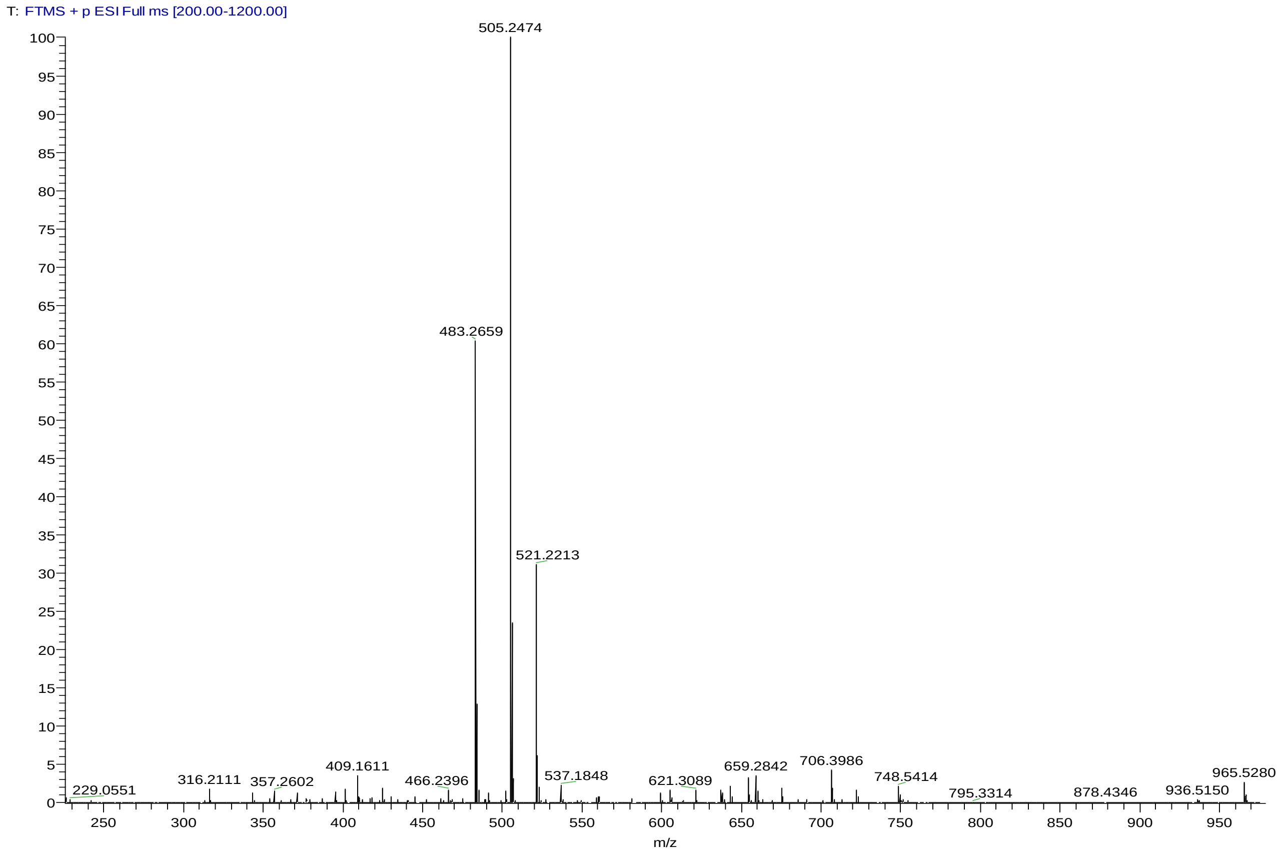

Datafile Name: Peptide 3

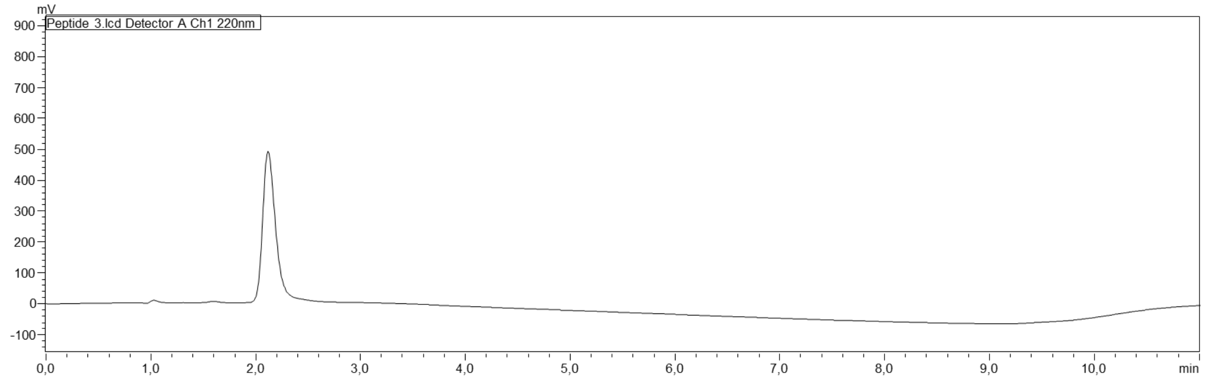

Figure S1. HR-ESI-MS of Peptide **3** ion $[M+H]^+$ and analytical HPLC trace at 220 nm.

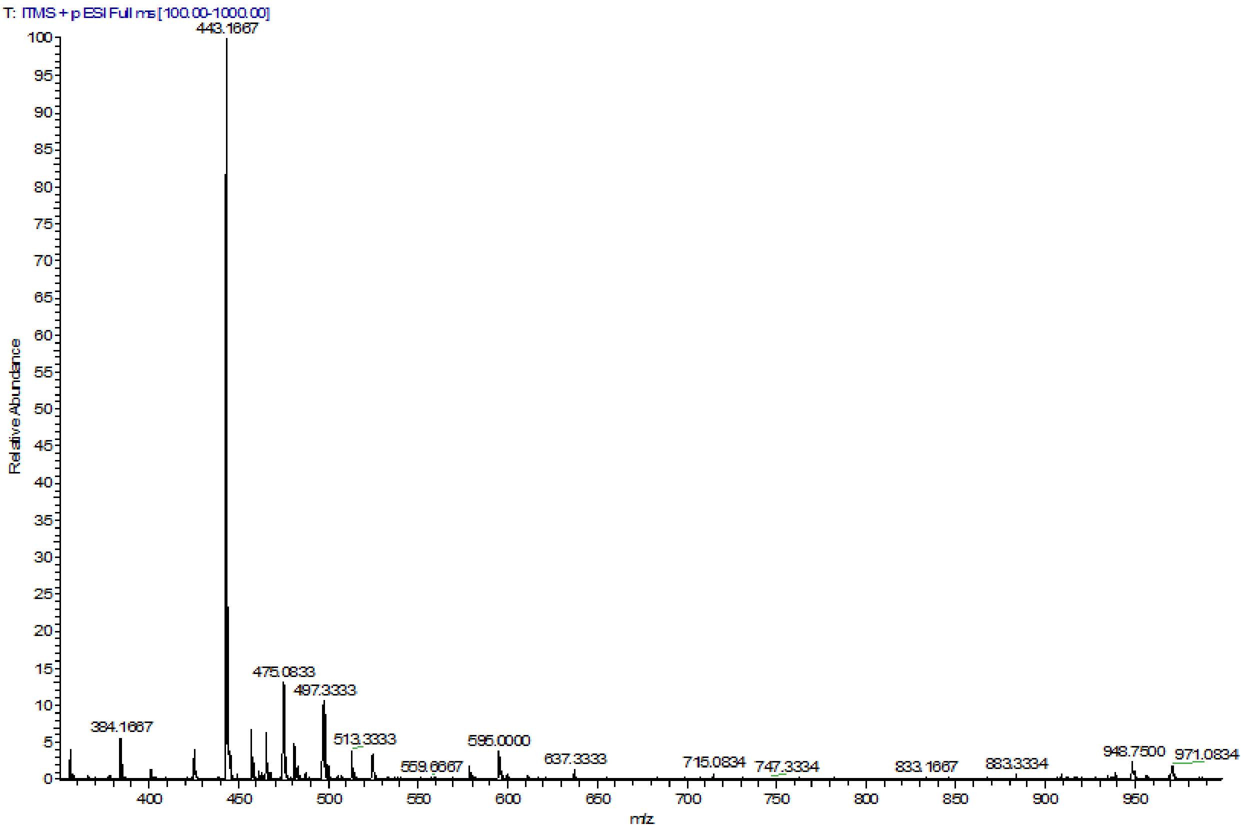

Datafile Name: Peptide 4

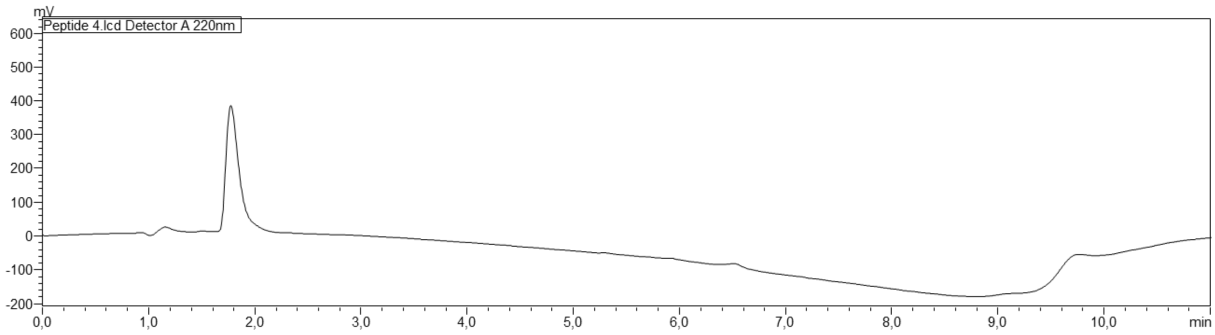

Figure S2. HR-ESI-MS of Peptide **4** ion $[M+H]^+$ and analytical HPLC trace at 220 nm.

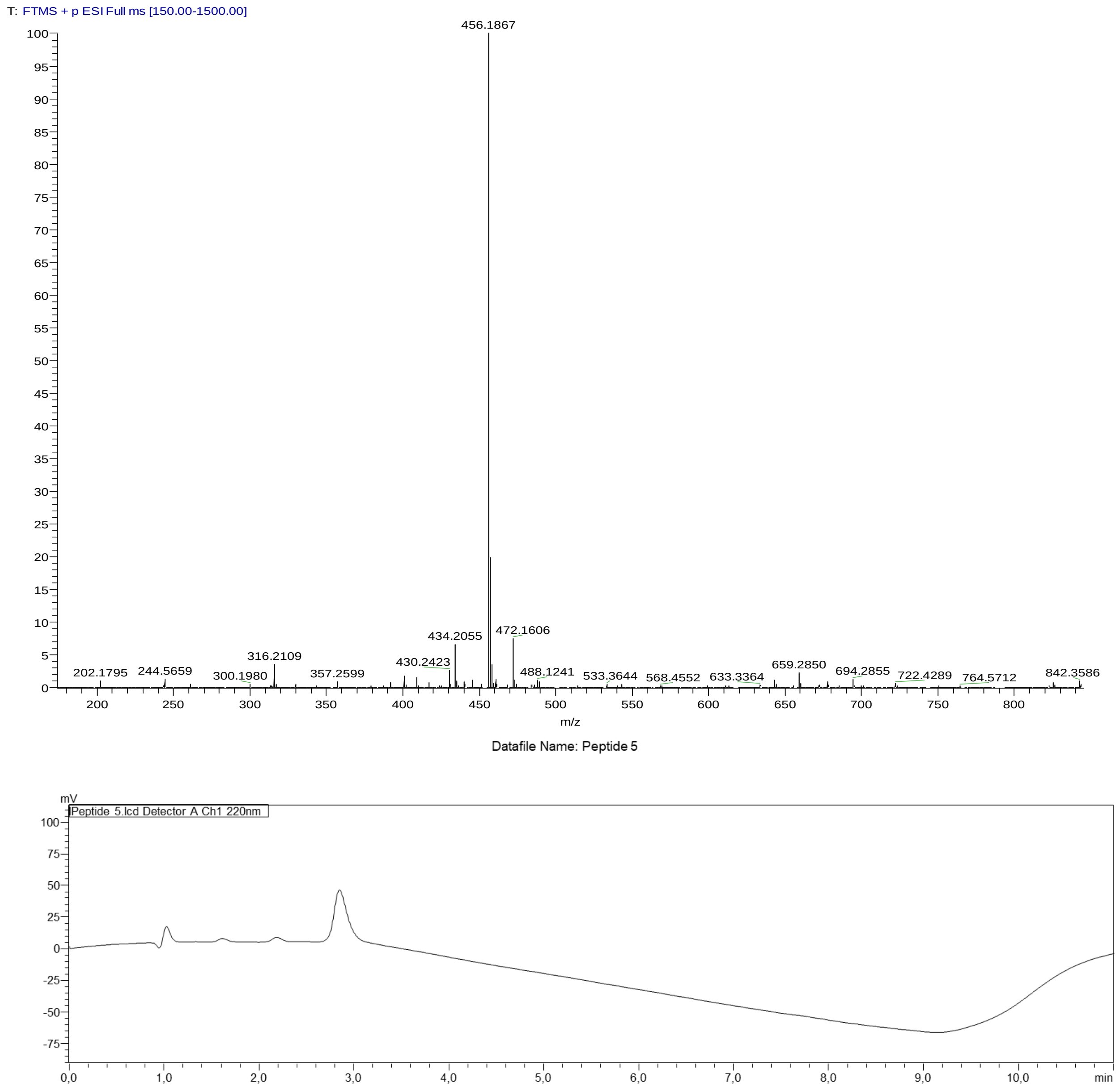

Figure S3. HR-ESI-MS of Peptide **5** ion $[M+H]^+$ and analytical HPLC trace at 220 nm.

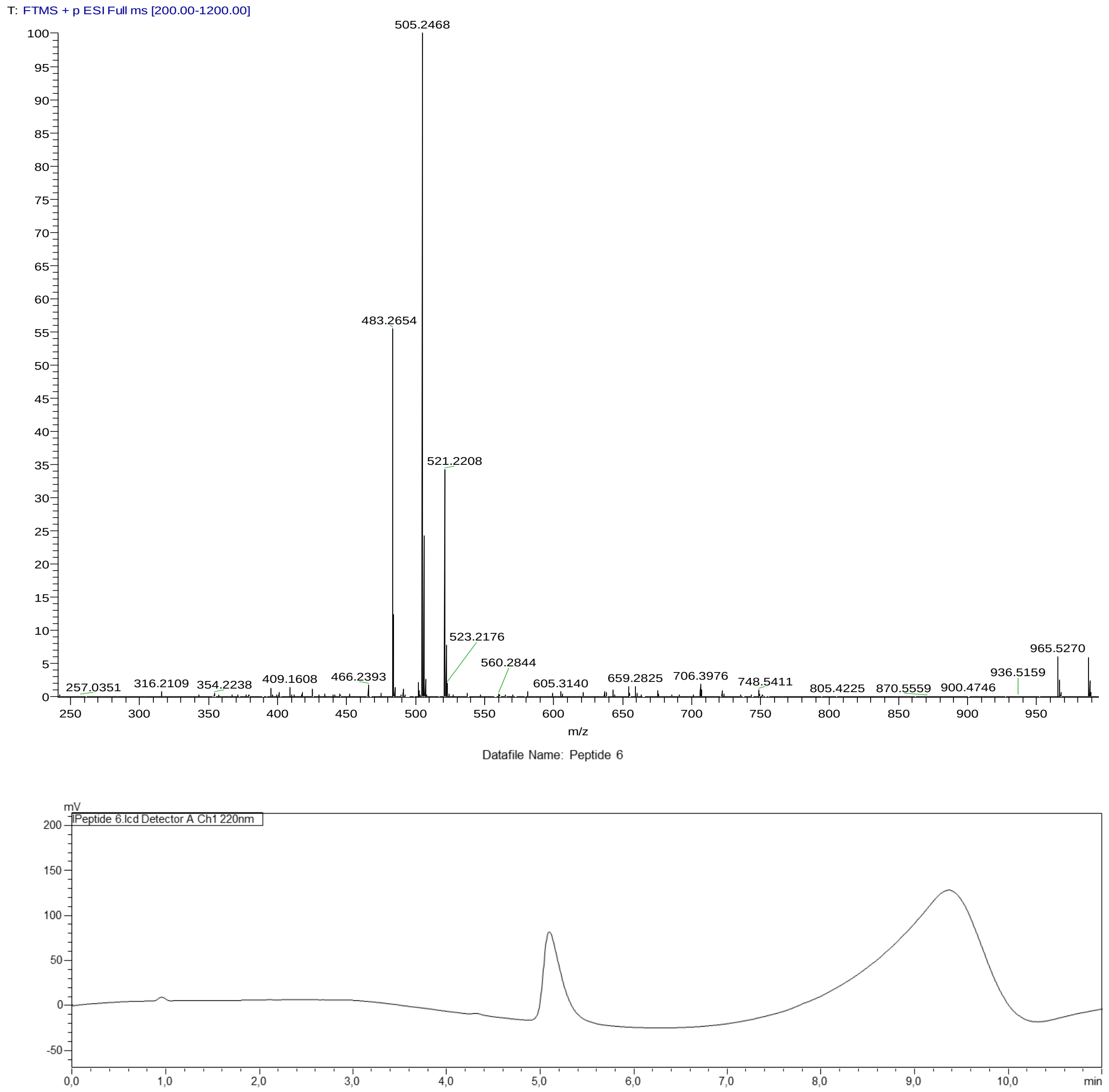

Figure S4. HR-ESI-MS of Peptide **6** ion $[M+H]^+$ and analytical HPLC trace at 220 nm.

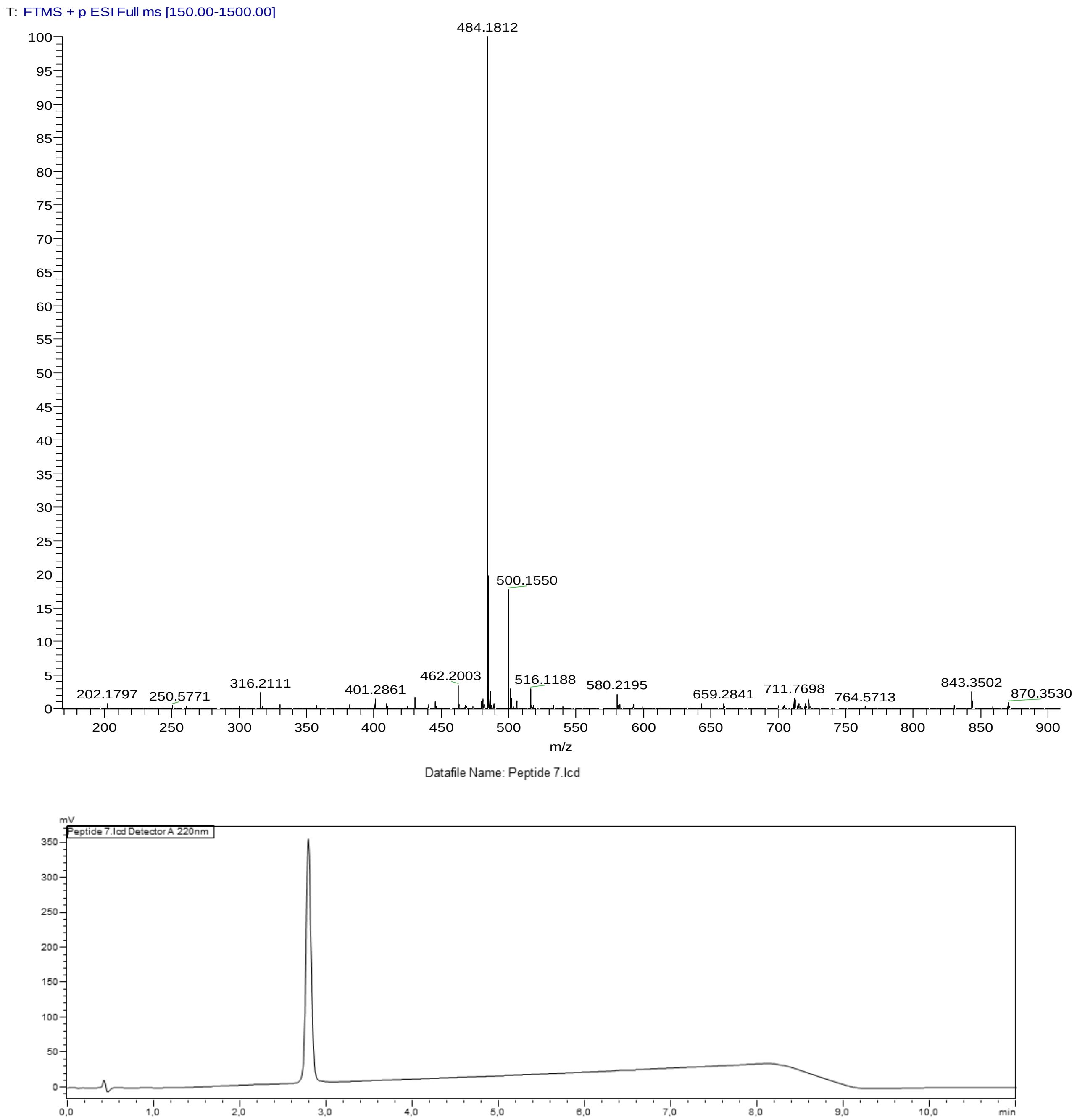

Figure S5. HR-ESI-MS of Peptide **7** ion $[M+H]^+$ and analytical HPLC trace at 220 nm.

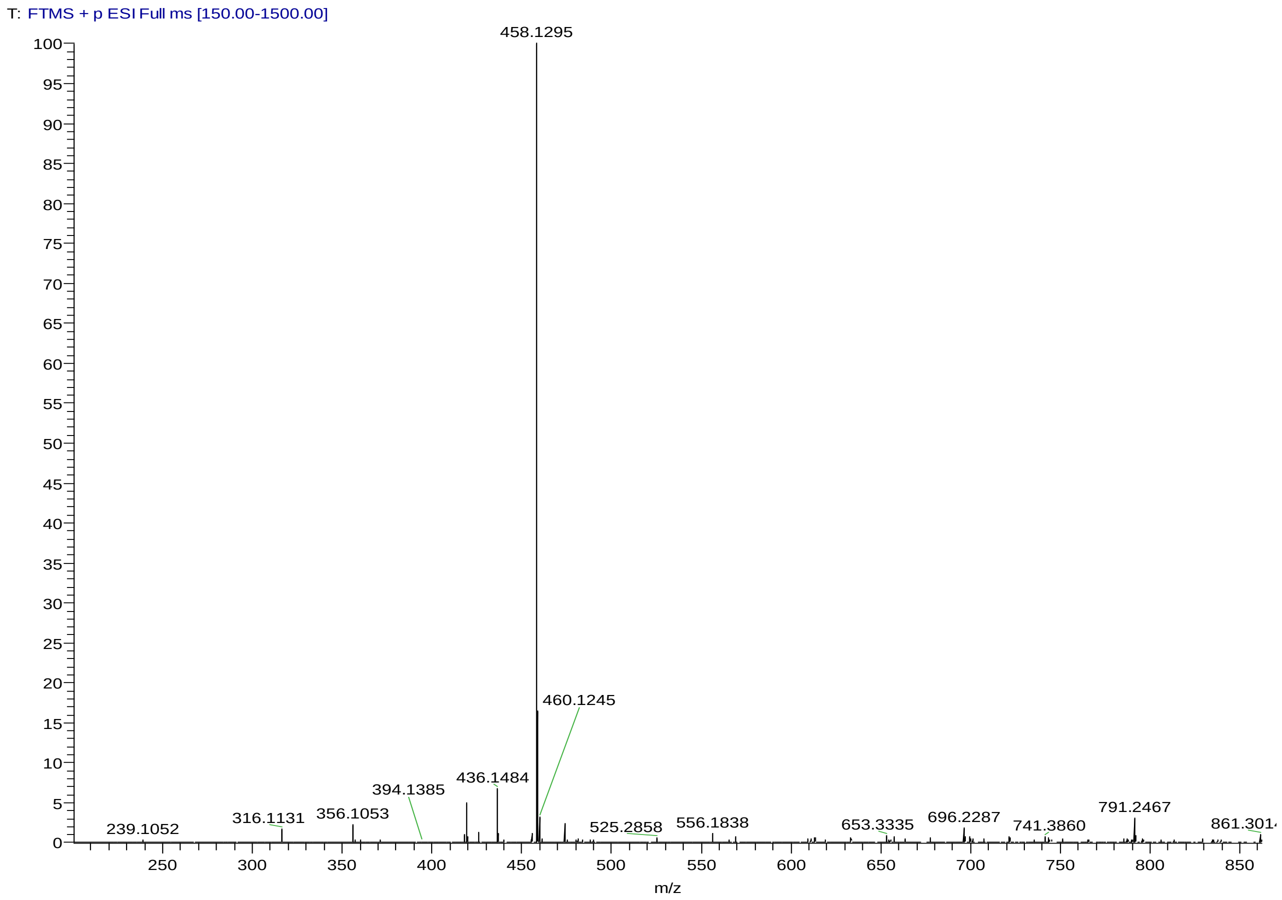

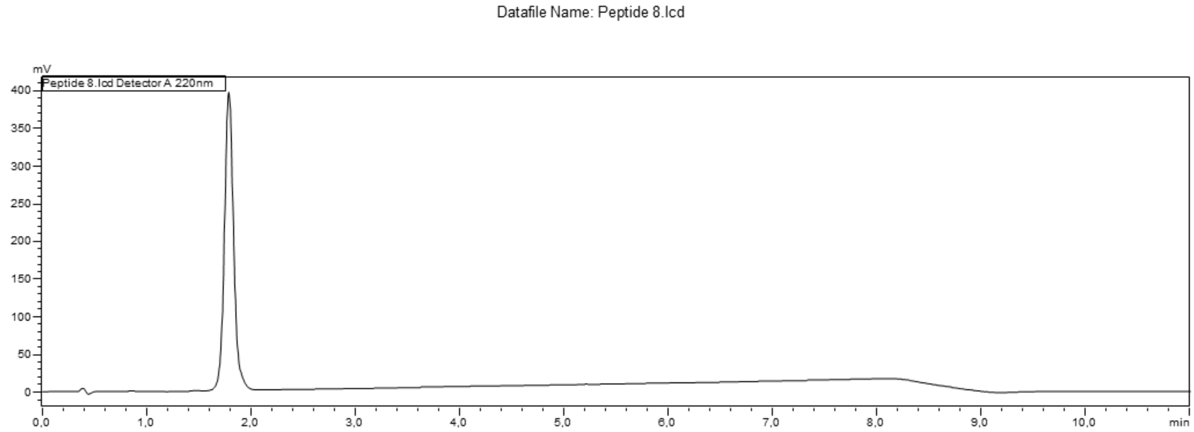

Figure S6. HR-ESI-MS of Peptide **8** ion $[M+H]^+$ and analytical HPLC trace at 220 nm.

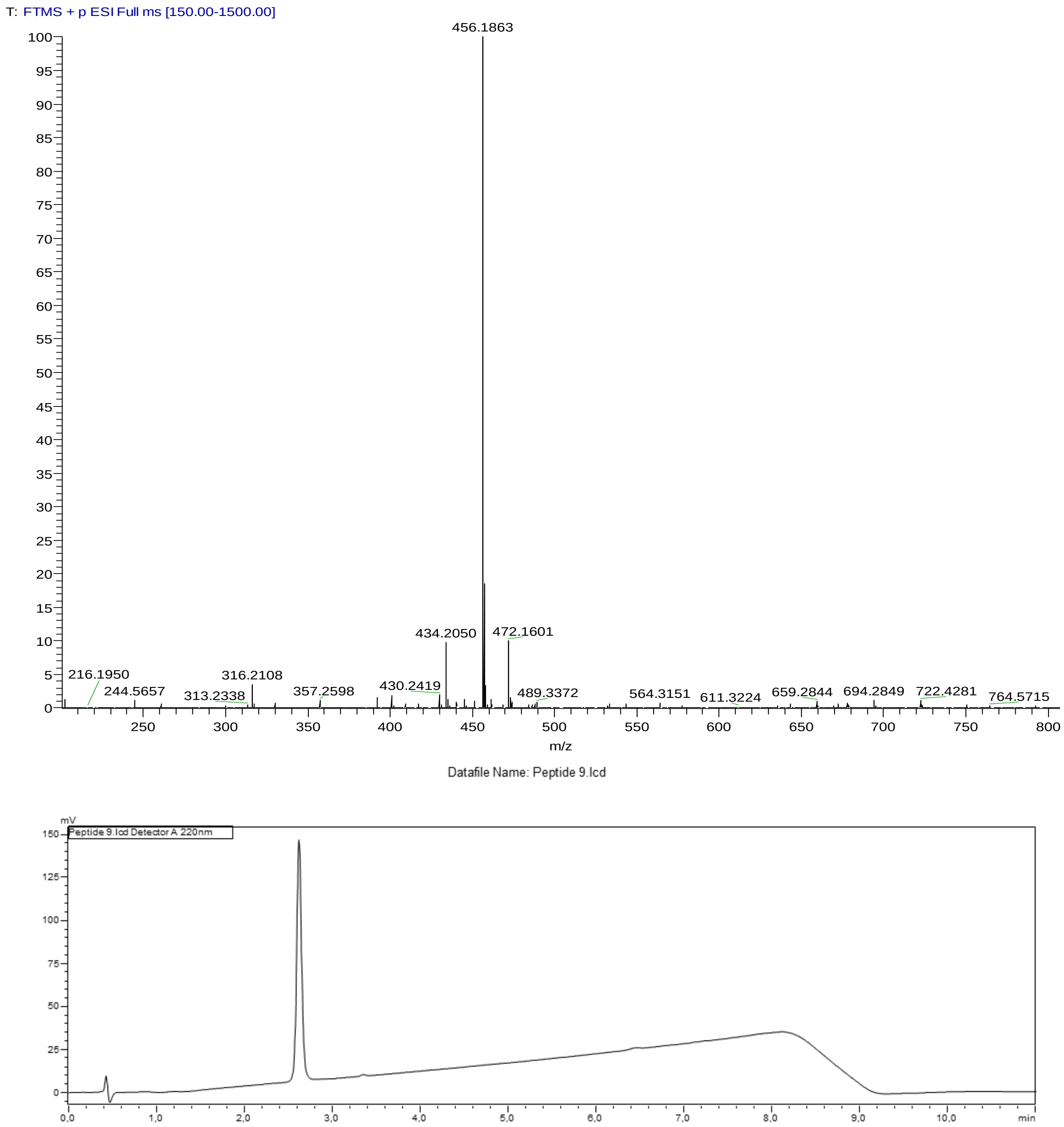

Figure S7. HR-ESI-MS of Peptide **9** ion $[M+H]^+$ and analytical HPLC trace at 220 nm.

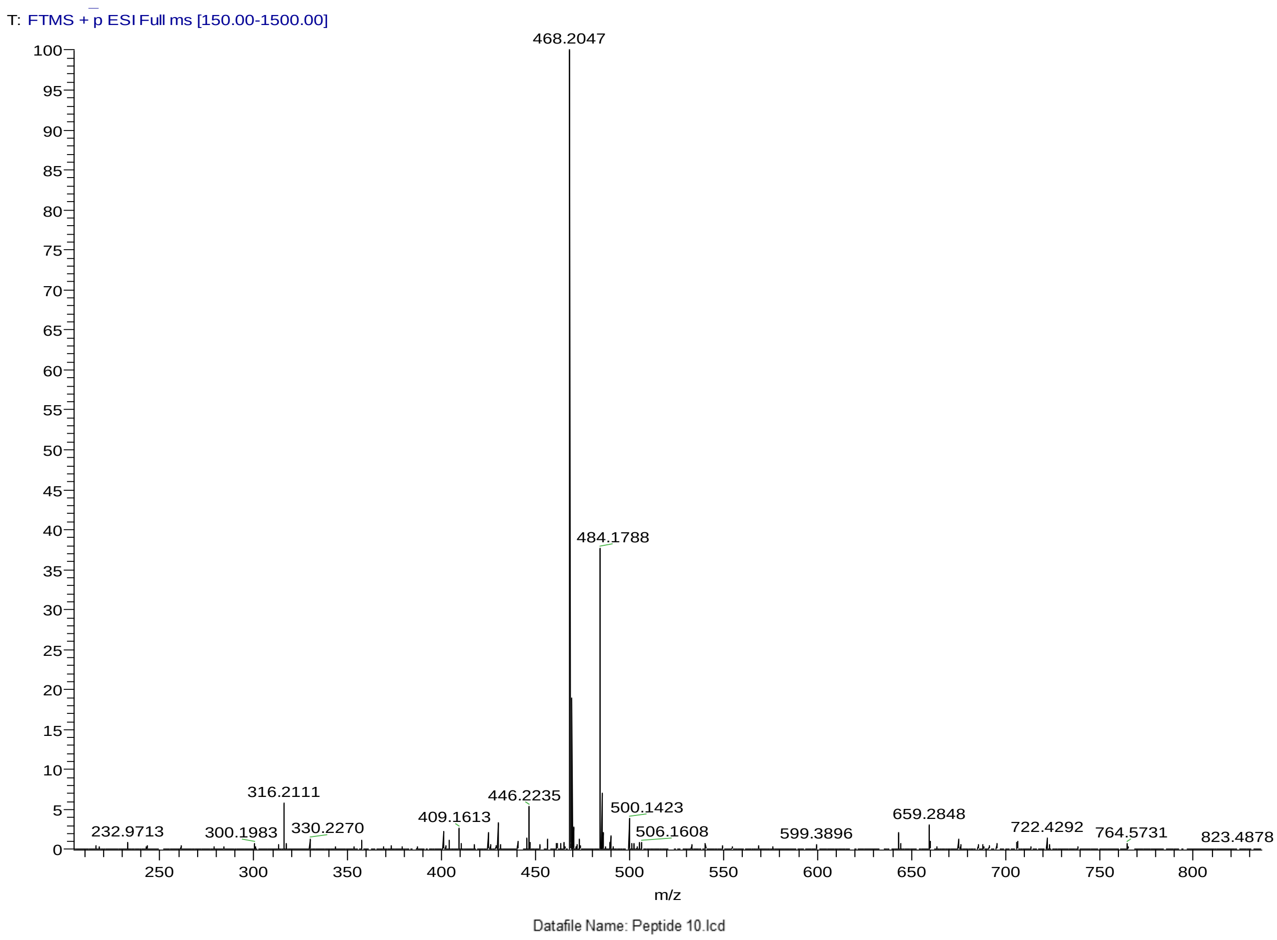

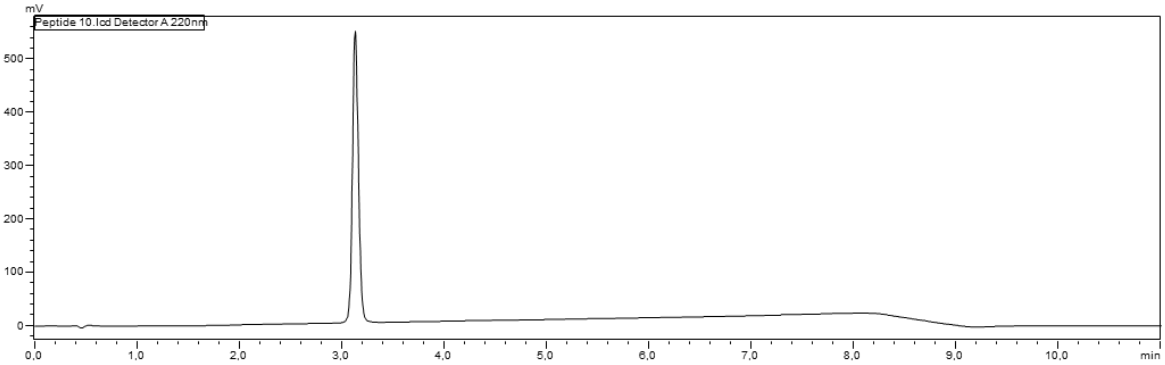

Figure S8. HR-ESI-MS of Peptide **10** ion $[M+H]^+$ and analytical HPLC trace at 220 nm.

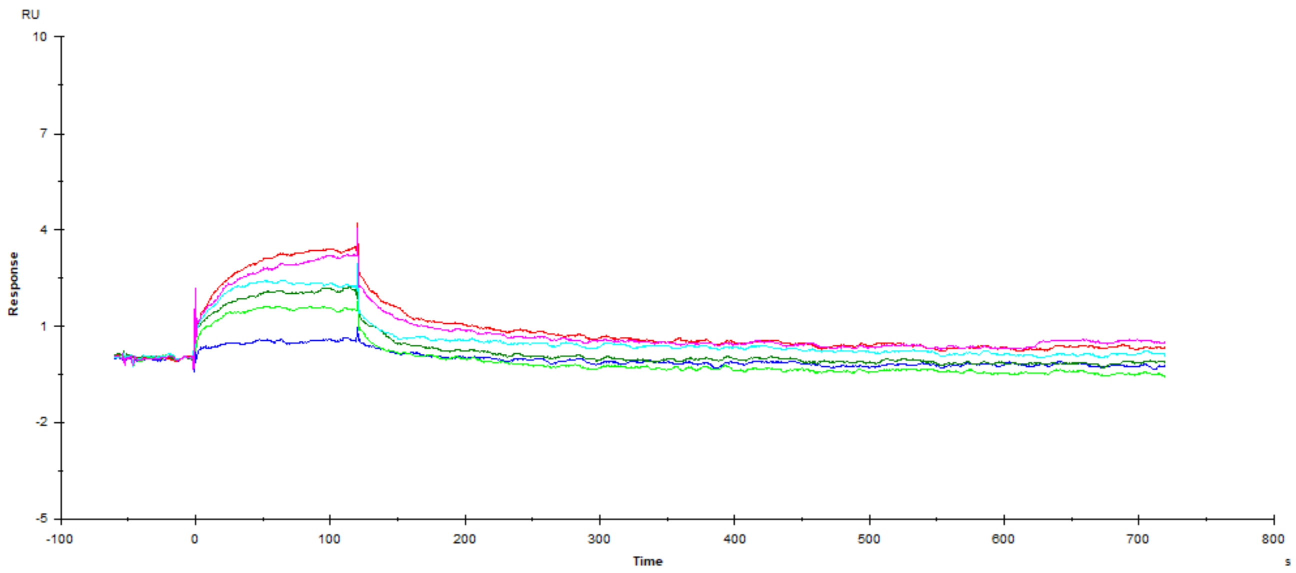

Figure S9. SPR sensorgrams between HA (immobilized on the sensor chip surface) and the compound **3**.

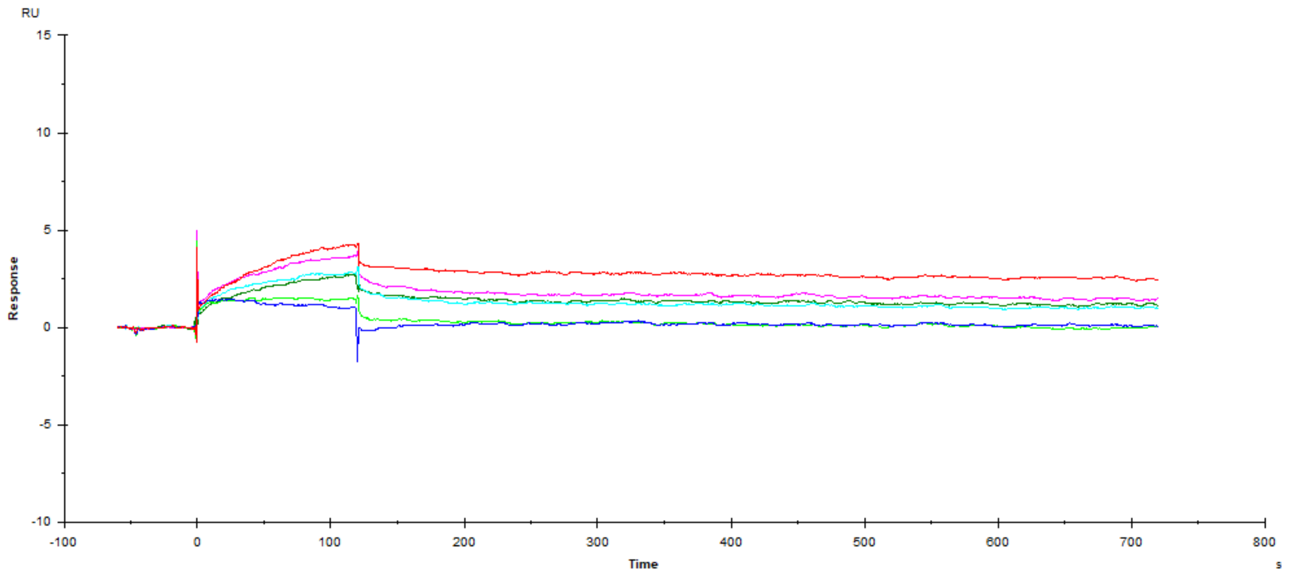

Figure S10. SPR sensorgrams between HA (immobilized on the sensor chip surface) and the compound **5**.

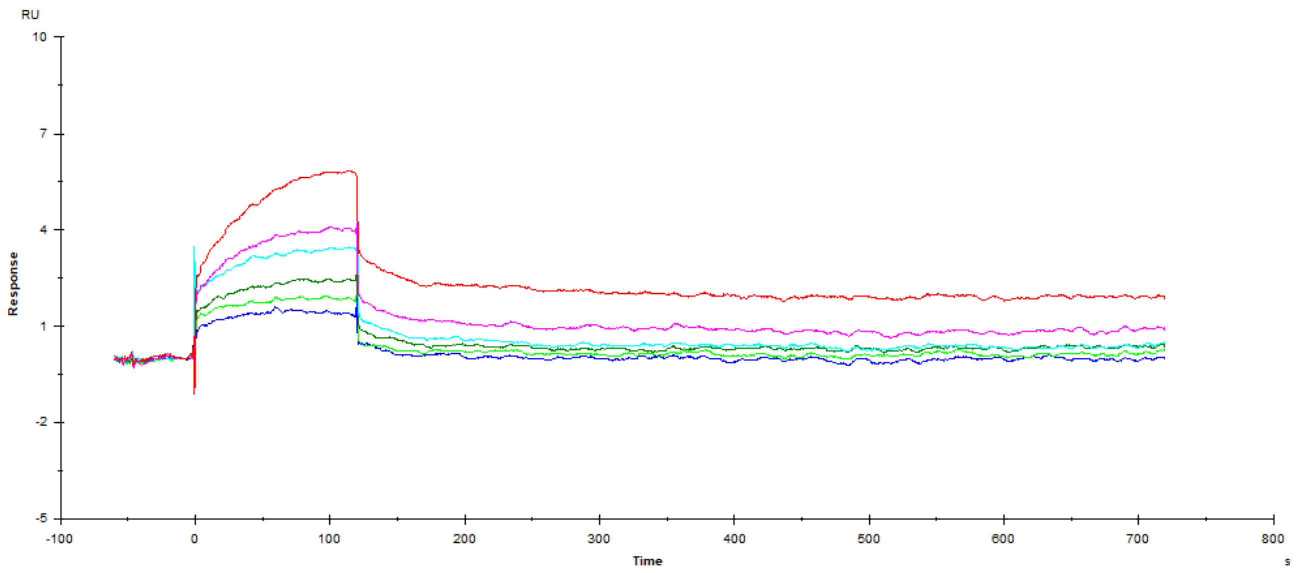

Figure S11. SPR sensorgrams between HA (immobilized on the sensor chip surface) and the compound **6**.

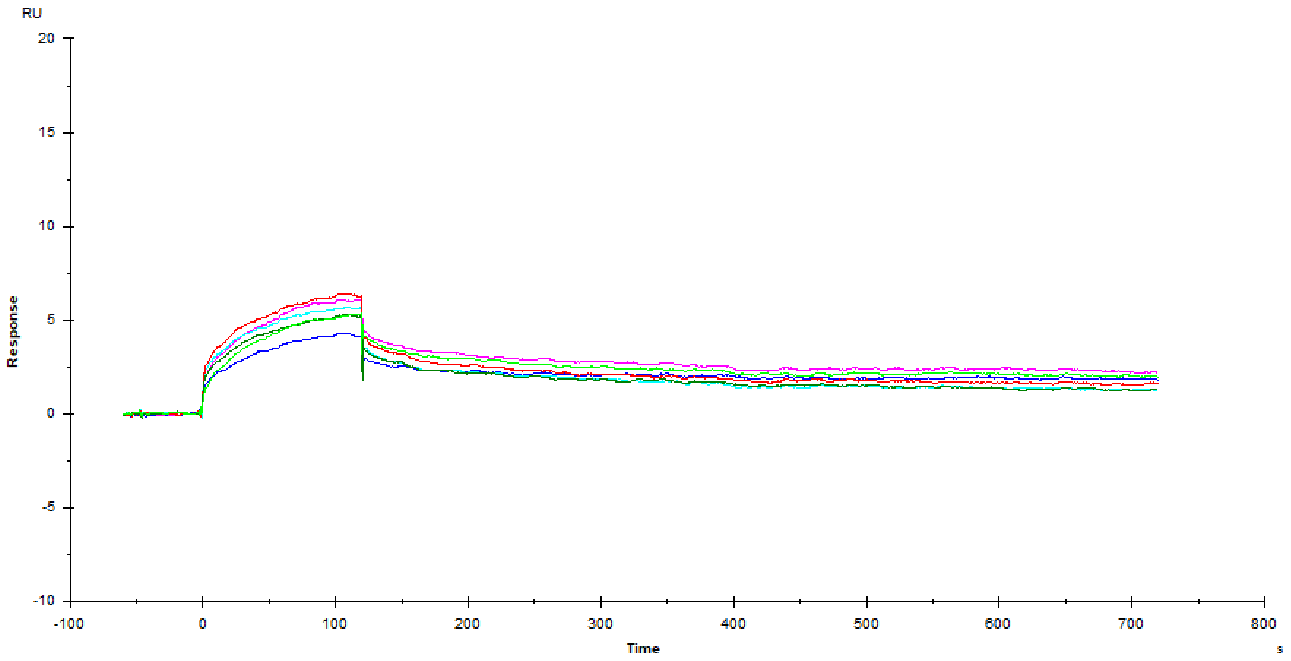

Figure S12. SPR sensorgrams between HA (immobilized on the sensor chip surface) and the compound **7**.

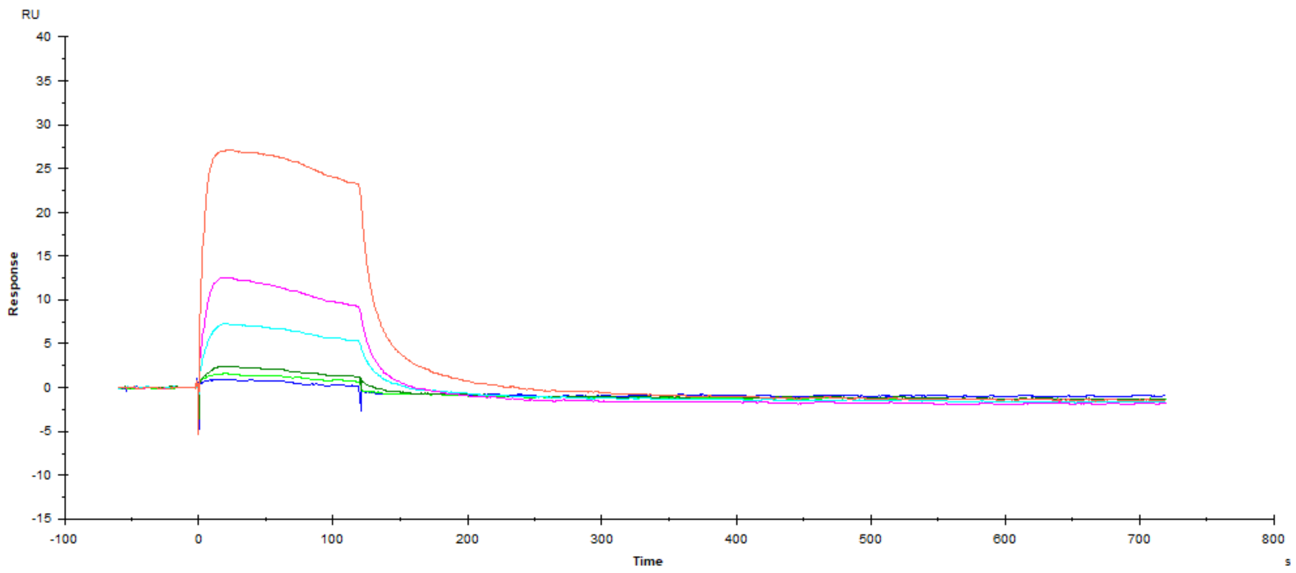

Figure S13. SPR sensorgrams between HA (immobilized on the sensor chip surface) and the compound **8**.

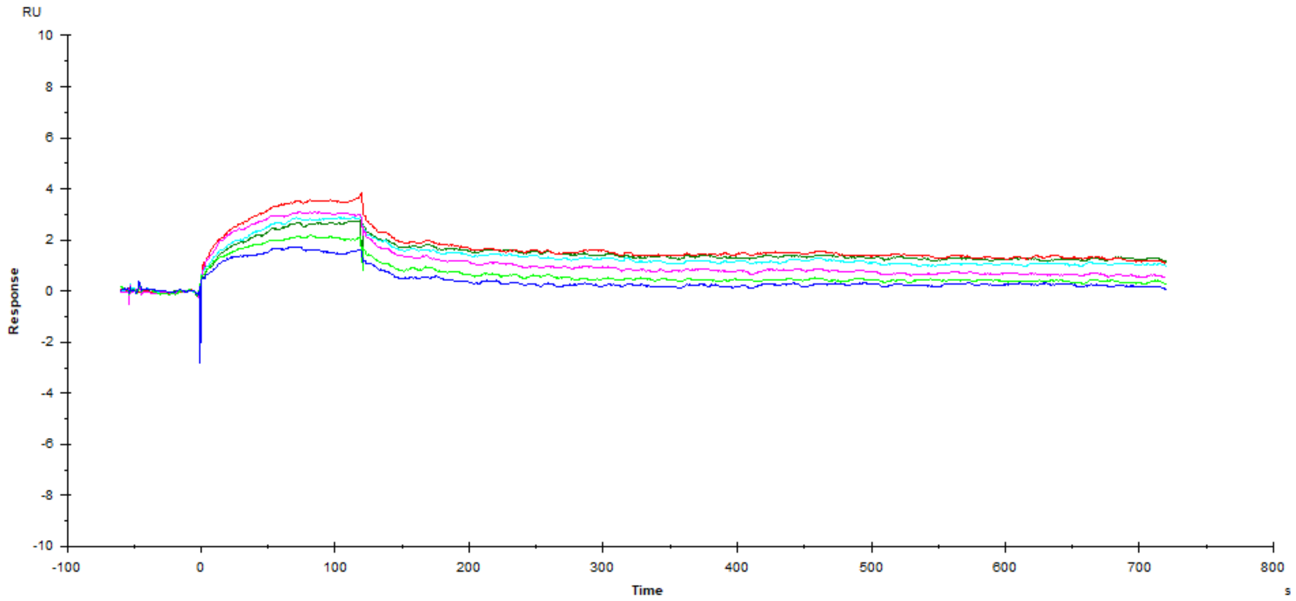

Figure S14. SPR sensorgrams between HA (immobilized on the sensor chip surface) and the compound **9**.

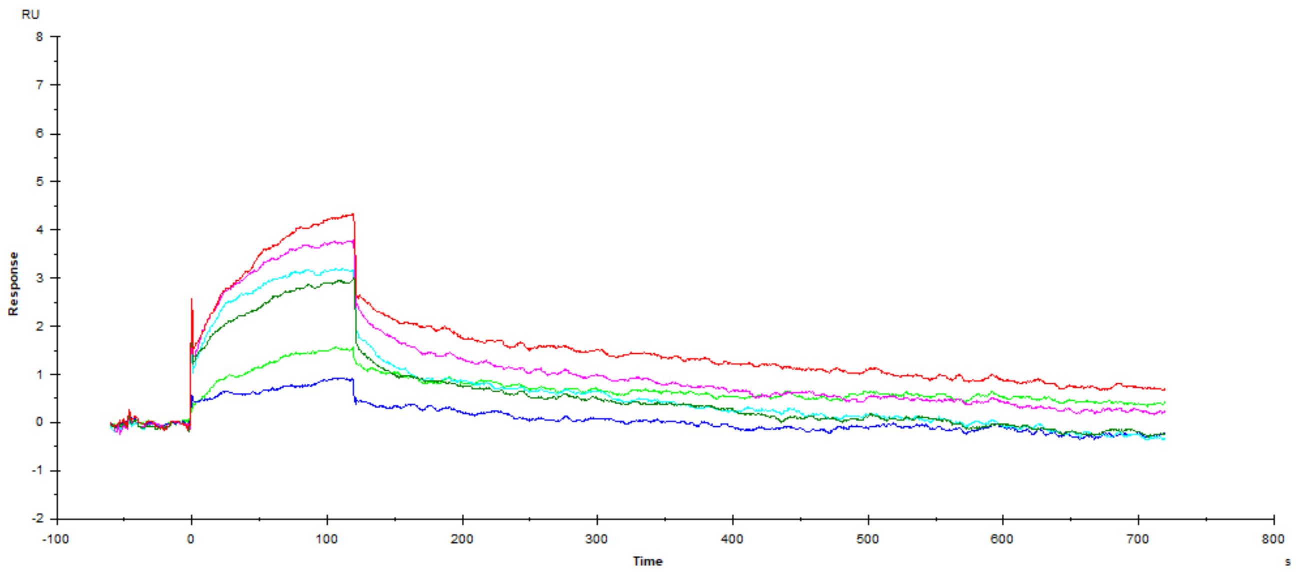

Figure S15. SPR sensorgrams between HA (immobilized on the sensor chip surface) and the compound **10**.

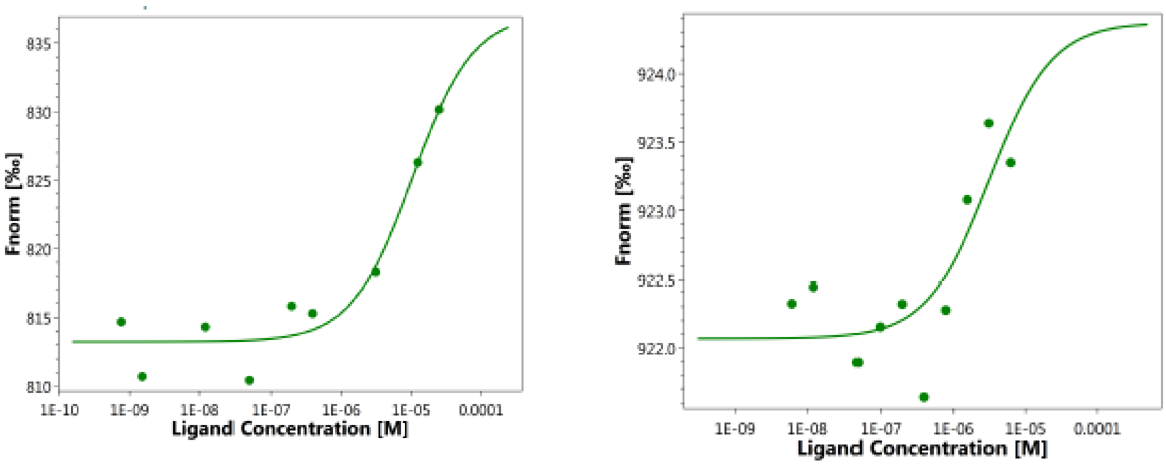

Figure S16. MST binding curve of peptide **2-3** to HA.

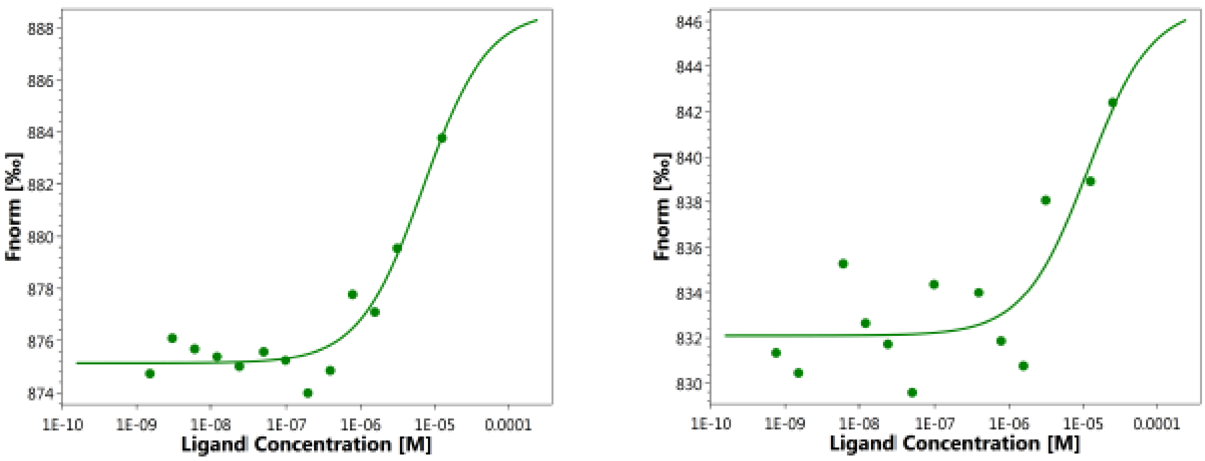

Figure S17. MST binding curve of peptide **5-6** to HA.

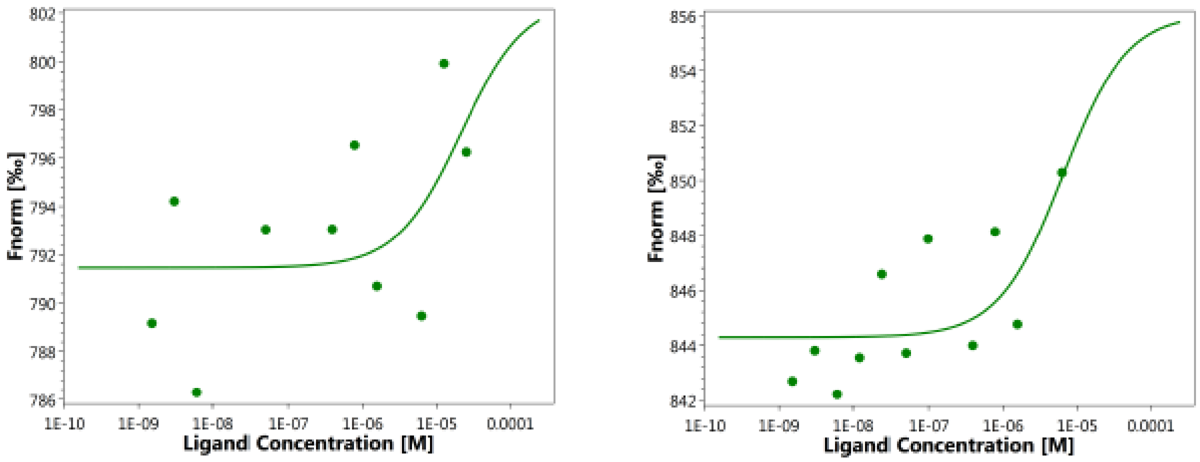

Figure S18. MST binding curve of peptide **7-8** to HA.

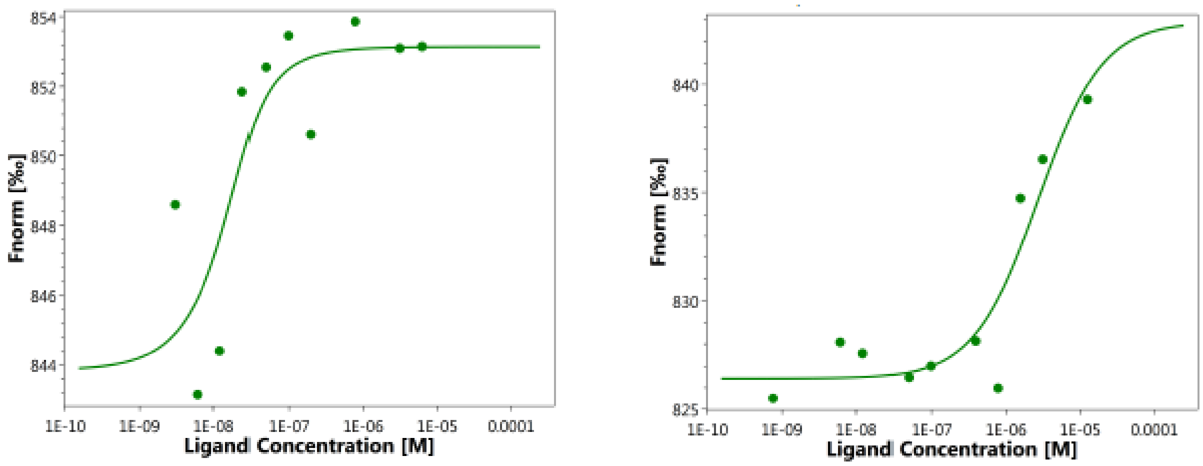

Figure S19. MST binding curve of peptide **9-10** to HA.

Table S2. Best ranking templates used for model generation in Swiss-Model for each HA

Structure	Template	Seq Identity	Oligo-state	QSQE	Found by	Method	Resolution	Seq Similarity	Coverage
Parma_H1N1	6wcr.1	85.92	hetero-3-3-mer	0.95	HHblits	X-ray	2.68Å	0.58	0.90
Roma_H1N1	6wcr.1	85.95	hetero-3-3-mer	0.96	HHblits	X-ray	2.68Å	0.58	0.90
Parma_H3N2	6aop.1	98.17	hetero-3-3-mer	0.97	HHblits	X-ray	2.30Å	0.62	0.90

Table S3. Score values obtained for the best homology models

Structure	Built with	Oligo-state	GMQE	QMEAN
Parma_H1N1	ProMod3 3.1.1	hetero-3-3-mer	0.85	-0.16
Roma_H1N1	ProMod3 3.1.1	hetero-3-3-mer	0.84	-0.08
Parma_H3N2	ProMod3 3.1.1	hetero-3-3-mer	0.90	-0.06

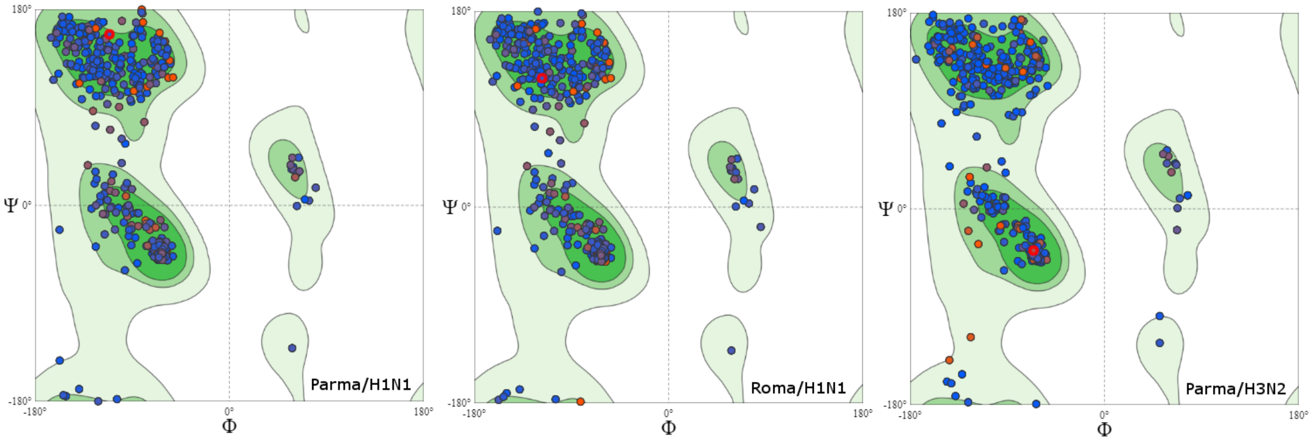

Figure S20. Ramachandran Plots obtained for HA homology models

```
H1N1_Parma  1    ------STDTVDTVLEKNVTVTHSVNLLENSHNGKLCLLKGIA
H1N1_Roma   1    ANNSTDTVDTVLEKNVTVTHSVNLLENSHNGKLCLLKGIA

H1N1_Parma  38   PLQLGNCSVAGWILGNPECELLISKESWSYIVEKPNPENG
H1N1_Roma   41   PLQLGNCSVAGWILGNPECELLISKESWSYIVEKPNPENG

H1N1_Parma  78   TCYPGHFADYEELREQLSSVSSFERFEIFPKESSWPNHTV
H1N1_Roma   81   TCYPGHFADYEELREQLSSVSSFERFEIFPKESSWPNHTV

H1N1_Parma  118  TGVS--SCSHNGENSFYRNLLWLTGKNGLYPNLSKSYANNK
H1N1_Roma   121  TGVSASCSHNGESSFYRNLLWLTGKNGLYPNLSKSYANNK

H1N1_Parma  157  EKEVLVLWGVHHPPNIADQKTLYHTENAYVSVVSSHYSRK
H1N1_Roma   161  EKEVLVLWGVHHPPNIGDQKALYHTENAYVSVVSSHYSRK

H1N1_Parma  197  FTPEIAKRPKVRDQEGRINYYWTLLEPGDTIIFEANGNLI
H1N1_Roma   201  FTPEIAKRPKVRDQEGRINYYWTLLEPRDTIIFEANGNLI

H1N1_Parma  237  APRYAFALSRGFGSGIINSNAPMDKCDAKCQTPQGAINSS
H1N1_Roma   241  APRYAFALSRGFGSGIINSNAPMDKCDAKCQTPQGAINSS

H1N1_Parma  277  LPFQNVHPVTIGECPKYVRSAKLRMVTGLRNIPSGLFGAI
H1N1_Roma   281  LPFQNVHPVTIGECPKYVRSAKLRMVTGLRNIPS-----GAI

H1N1_Parma  7    AGFIEGGWTGMVDGWYGYHHQNEQGSGYAADQKSTQNAIN
H1N1_Roma   4    AGFIEGGWTGMVDGWYGYHHQNEQGSGYAADQKSTQNAIN

H1N1_Parma  47   GITNKVNSVIEKMNTQFTAVGKEFNKLERRMENLNKKVDD
H1N1_Roma   44   GITNKVNSVIEKMNTQFTAVGKEFNKLERRMENLNKKVDD

H1N1_Parma  87   GFIDIWTYNAELLVLLENERTLDFHDSNVKNLYEKVKSQL
H1N1_Roma   84   GFIDIWTYNAELLVLLENERTLDFHDSNVKNLYEKVKSQL

H1N1_Parma  127  KNNAKEIGNGCFEFYHKCNDECMESVKNGTYDYPKYSEES
H1N1_Roma   124  KNNAKEIGNGCFEFYHKCNDECMESVKNGTYDYPKYSEES

H1N1_Parma  167  KLNRE------STDTVDTVLEKNVTVTHSVNLLENSHNGKLCL
H1N1_Roma   164  KLNREANNSTDTVDTVLEKNVTVTHSVNLLENSHNGKLCL
```

Figure S21. Alignment of HA sequences of A/Parma and A/Roma H1N1 strains

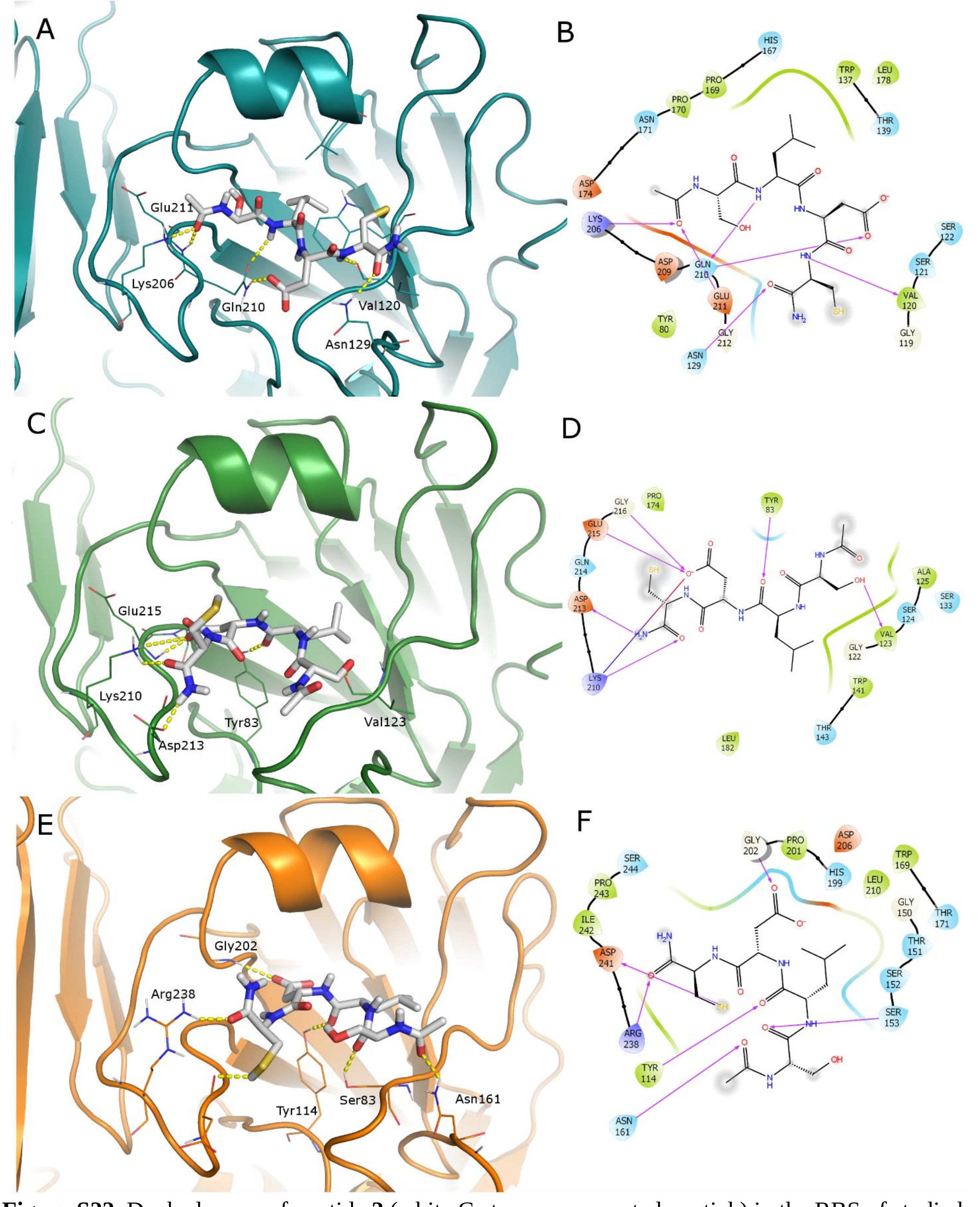

Figure S22. Docked poses of peptide **2** (white C atoms, represented as stick) in the RBS of studied HAs represented as cartoon: A) Parma/H1N1 (deep-cyan); B) Roma/H1N1 (dark green); C) Parma/H3N2 (orange). Residues involved in H-bond interactions with the ligand are represented as lines; H-bond are depicted as yellow dashed lines. Corresponding 2D ligand interaction diagrams are reported in panels B), D) and E); in the diagrams residues close to the ligand are coloured on the basis of their properties (orange, negatively charged; blue, positively charged; green, hydrophobic; cyan, polar), H-bonds are depicted as magenta arrows, solvent exposed atoms are surrounded by a grey shadow.

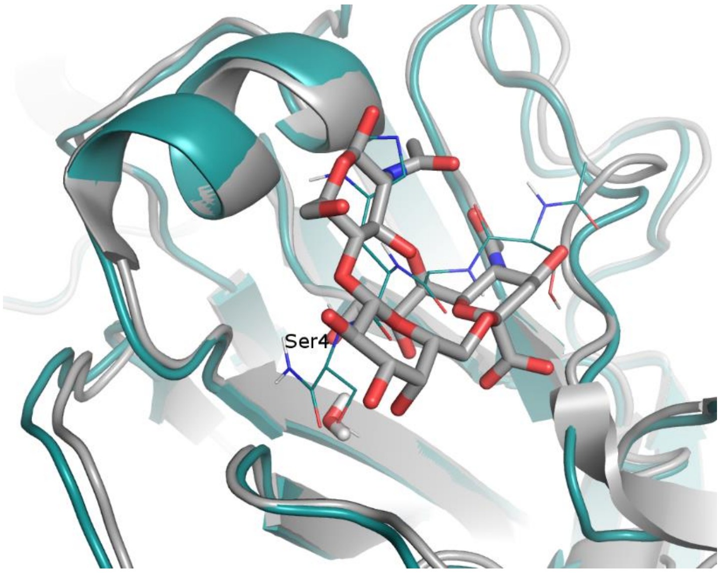

Figure S23. Superposition of the complex of HA:sialic acid (PDB ID 3UBN, grey carbon atoms) with docked pose of SAHS in the A/Parma/24/09 H1N1 (deepcyan carbon atoms). Proteins are represented as cartoon. Sialic acid and conserved water molecule are represented as stick, while the SAHS is represented as thin stick.
